# Supplementary material for: Problem-solving training: assessing the feasibility and acceptability of delivering and evaluating a problem-solving training model for front-line prison staff and prisoners who self-harm
Source: BMJ Open. 2019 Oct 3;9(10):e026095. doi: 10.1136/bmjopen-2018-026095 (PMC6797432; doi:10.1136/bmjopen-2018-026095)
Supplement: Supplementary data [file bmjopen-2018-026095supp001.pdf]

Web Appendix Supplementary Materials

Appendix A: Table one data collection protocols

Appendix B: Table two prison staff focus group participation

Appendix C: Table three prisoner focus group participation

Appendix D: Table four prisoner self-harm details

Appendix E: Table five coding variability across prison sites

Appendix F: Figure 1 numbers of prisoners at unlock on last day of the month

Appendix G: Table six standardised ACCT process costs

Appendix H: Table seven training and implementation costs

Appendix I: Figure 2 prisoner ACCTs, intervention and post assessment relative to baseline assessment

Appendix J: Figure 3 Phq-9 score at baseline and follow-up

Perry A, *et al. BMJ Open* 2019; 9:e026095. doi: 10.1136/bmjopen-2018-026095

Appendix A: Table one data collection protocols

Prison Site	Prison A	Prison B	Prison D	Prison C
**Frequency of ACCTs per year**				
2009			61	
2010			118	
2011	754		168	
2012	756	840	170	779
2013	730	734	154	718
2014	1012	645	208	688
2015	1219	798	249	729
2016 – partial*	1010	675	262	605
**Number of ACCTs per year (2012 - 2015)**				
Mean (SD)	929.3 (231.31)	754.3 (84.88)	195.3 (42.39)	728.5 (37.86)
Median (Range)	884 (730, 1219)	766 (645, 840)	189 (154, 249)	724 (688, 779)

*2016 frequencies up to September in Prison A, D and Cl, and up to November in Prison B.

Appendix B: Table two prison staff focus group participation

	PrisonA (n=10)	PrisonB (n=11)	PrisonC (n=10)
**Type of staff**			
Operational staff	7 (70.0%)	7 (63.6%)	3 (30.0%)
Managerial staff	0 (0.0%)	1 (9.1%)	5 (50.0%)
Healthcare staff	0 (0.0%)	1 (9.1%)	2 (20.0%)
Visitor to the prison	2 (20.0%)	0 (0.0%)	0 (0.0%)
Admin / Probation	1 (10.0%)	1 (9.1%)	0 (0.0%)
Missing	0 (0.0%)	1 (9.1%)	0 (0.0%)
**Gender**			
Male	4 (40.0%)	5 (45.5%)	3 (30.0%)
Female	6 (60.0%)	6 (54.5%)	7 (70.0%)
**Age**			
N	10	10	9
Mean (SD)	33.7 (10.07)	42.7 (11.41)	46.9 (6.15)
Median (Range)	33.0 (19, 49)	44.0 (21, 58)	47.0 (36, 56)
**First language**			
English	8 (80.0%)	11 (100.0%)	10 (100.0%)
Hungarian	0 (0.0%)	0 (0.0%)	0 (0.0%)
German	0 (0.0%)	0 (0.0%)	0 (0.0%)
Missing	2 (20.0%)	0 (0.0%)	0 (0.0%)
**Ethnic group**			
British	10 (100.0%)	11 (100.0%)	9 (90.0%)
Irish / Other white background	0 (0.0%)	0 (0.0%)	0 (0.0%)
White and Black Caribbean	0 (0.0%)	0 (0.0%)	0 (0.0%)
Indian / Pakistani	0 (0.0%)	0 (0.0%)	1 (10.0%)
**Religious preference**			
No religion	2 (20.0%)	6 (54.5%)	1 (10.0%)
Christian	8 (80.0%)	5 (45.5%)	8 (80.0%)
Muslim	0 (0.0%)	0 (0.0%)	1 (10.0%)
Hindu	0 (0.0%)	0 (0.0%)	0 (0.0%)
**Consider yourself disabled?**			
Yes	0 (0.0%)	0 (0.0%)	0 (0.0%)
No	10 (100.0%)	11 (100.0%)	10 (100.0%)
**Highest academic qualification?**			
Post Graduate	0 (0.0%)	0 (0.0%)	0 (0.0%)
Graduate	5 (50.0%)	5 (45.5%)	2 (20.0%)
A Level or equivalent	5 (50.0%)	3 (27.3%)	7 (70.0%)
GCSE or equivalent	0 (0.0%)	3 (27.3%)	1 (10.0%)

Perry A, *et al. BMJ Open* 2019; 9:e026095. doi: 10.1136/bmjopen-2018-026095

Appendix C: Table three prisoner focus group participation

	PrisonA (n=13)	PrisonB (n=43)	PrisonC (n=11)	Total (n=67)
**Are you on Remand?**				
Yes	2 (15.4%)	2 (4.7%)	1 (9.1%)	5 (7.5%)
No	11 (84.6%)	38 (88.4%)	10 (90.9%)	59 (88.1%)
Missing	0 (0.0%)	3 (7.0%)	0 (0.0%)	3 (4.5%)
**First time offender?**				
Yes	4 (30.8%)	19 (44.2%)	7 (63.6%)	30 (44.8%)
No	9 (69.2%)	23 (53.5%)	4 (36.4%)	36 (53.7%)
Missing	0 (0.0%)	1 (2.3%)	0 (0.0%)	1 (1.5%)
**Age first entered prison (years)**				
N	13	42	11	66
Mean (SD)	21.3 (7.61)	30.0 (13.13)	40.5 (12.71)	30.0 (13.35)
Median (Range)	18.0 (15.0, 40.0)	27.0 (13.0, 61.0)	39.0 (18.0, 60.0)	26.0 (13.0, 61.0)
**Number of times in prison?**				
N	13	40	11	64
Mean (SD)	7.2 (8.67)	2.8 (3.12)	1.2 (0.40)	3.4 (4.94)
Median (Range)	4.0 (0.0, 30.0)	1.5 (1.0, 15.0)	1.0 (1.0, 2.0)	1.0 (0.0, 30.0)
**Time spent in this prison? (months)**				
N	13	42	11	66
Mean (SD)	9.2 (10.66)	29.0 (25.27)	34.8 (26.98)	26.0 (24.71)
Median (Range)	6.0 (1.0, 42.0)	24.0 (2.0, 102.0)	24.0 (11.0, 84.0)	16.0 (1.0, 102.0)
**Length of sentence (months)**				
N	11	38	11	60
Mean (SD)	167.5 (344.02)	208.5 (231.48)	181.9 (108.94)	196.1 (236.56)
Median (Range)	30.0 (18, 1188)	120.0 (8, 666)	168.0 (42, 333)	126.0 (8, 1188)
**Months left until sentence expiry**				
N	9	30	8	47
Mean (SD)	36.1 (44.62)	64.0 (59.95)	76.5 (56.46)	60.8 (57.15)
Median (Range)	16.3 (3.7, 120.2)	41.0 (0.8, 257.9)	56.4 (6.2, 154.6)	40.2 (0.8, 257.9)

Perry A, *et al. BMJ Open* 2019; 9:e026095. doi: 10.1136/bmjopen-2018-026095

Appendix D: Table four prisoner self-harm details

	PrisonA (n=26)	PrisonB (n=18)	Prison D (n=4)	Total (n=48)
**Ever harmed yourself?**				
Yes	26 (100.0%)	18 (100.0%)	4 (100.0%)	48 (100.0%)
**Time since most recent self-harm (months)**				
N	26	16	4	46
Mean (SD)	1.0 (0.72)	0.7 (0.45)	1.5 (1.86)	1.0 (0.79)
Median (Range)	1.0 (0.0, 3.0)	0.7 (0.0, 1.5)	0.8 (0.1, 4.2)	0.7 (0.0, 4.2)
**Self-Harm frequency**				
Every day	1 (3.8%)	0 (0.0%)	0 (0.0%)	1 (2.1%)
Twice a week	3 (11.5%)	2 (11.1%)	0 (0.0%)	5 (10.4%)
Once a week	4 (15.4%)	1 (5.6%)	1 (25.0%)	6 (12.5%)
Every two weeks	0 (0.0%)	4 (22.2%)	1 (25.0%)	5 (10.4%)
Once a month	3 (11.5%)	2 (11.1%)	0 (0.0%)	5 (10.4%)
3 monthly	3 (11.5%)	1 (5.6%)	1 (25.0%)	5 (10.4%)
Less often than three monthly	12 (46.2%)	7 (38.9%)	1 (25.0%)	20 (41.7%)
Missing	0 (0.0%)	1 (5.6%)	0 (0.0%)	1 (2.1%)
**Type of most recent self-harm**				
Ligature	2 (7.7%)	3 (16.7%)	0 (0.0%)	5 (10.4%)
Cutting	14 (53.8%)	11 (61.1%)	4 (100.0%)	29 (60.4%)
OD Medication/ Recreational drug overdose	6 (23.1%)	4 (22.2%)	0 (0.0%)	10 (20.8%)
Electrocution	1 (3.8%)	0 (0.0%)	0 (0.0%)	1 (2.1%)
Hunger strike	3 (11.5%)	0 (0.0%)	0 (0.0%)	3 (6.3%)
**How easy was it for you to get help?**				
Very Easy	6 (23.1%)	4 (22.2%)	0 (0.0%)	10 (20.8%)
Took some time	10 (38.5%)	6 (33.3%)	2 (50.0%)	18 (37.5%)
There was no help available	7 (26.9%)	4 (22.2%)	0 (0.0%)	11 (22.9%)
I didn't bother to ask	3 (11.5%)	3 (16.7%)	2 (50.0%)	8 (16.7%)
can’t remember	0 (0.0%)	1 (5.6%)	0 (0.0%)	1 (2.1%)

Perry A, *et al. BMJ Open* 2019; 9:e026095. doi: 10.1136/bmjopen-2018-026095

Appendix E: Table five coding variability across prison sites

Prison B	Prison A	Prison C	Prison D
**Prior to Nov 2013:** Not available<br>**Nov 2013 – Aug 2014:** coded as self-harm or concerns<br>**Aug 2014 – Oct 2015:** free text reasons<br>**Oct 2015 – present:**<br>1. Suicide attempt or statement of intent to take own life<br>2. Self-injury or statement to self-harm<br>3. Unusual behaviour/talk<br>4. Very low mood<br>5. Drug Alcohol Withdrawal<br>6. Other concerns<br>7. Self-harm warning received from court<br>*Not possible to distinguish acts of self-harm and suicide from intent, statements, or concerns.*	Recorded as free text.<br><br>*Not always possible to distinguish acts of self-harm and suicide from intent, statements, or concerns.*	Not available	**Prior to mid-2016:**<br>1. Suicide Attempt<br>2. Statement/thoughts of intent to kill self<br>3. Self-Harm<br>4. Statement of intent/thoughts to self-harm<br>5. Unusual Behaviour<br>6. Low mood<br>7. Problems related to Drug / Alcohol withdraw<br>8. External Concerns<br>9. Deportation<br>10. Bullying<br>11. Other<br>**Mid-2016 to present:**<br>1. Suicide attempt or Statement of intent to take own life<br>2. Self-Injury or Statement to Self-Harm<br>3. Unusual behaviour/Talk<br>4. Low Mood<br>5. Drug Alcohol Withdrawal<br>6. Other Concerns<br>*Method coding also provided and varies*
**Following coding:**<br>• 39% Related to SH or suicide<br>• 20% Other<br>• 41% Missing	**Following coding:**<br>• 36% due to SH incident or suicide attempt<br>• 39% related to SH or suicide<br>• 25% Other<br>• <1% Missing		**Following coding:**<br>• 39% due to a SH incident or suicide attempt<br>• 24% related to SH or suicide<br>• 33% Other<br>• 4% Missing

Perry A, *et al. BMJ Open* 2019; 9:e026095. doi: 10.1136/bmjopen-2018-026095

Appendix F: Figure 1 numbers of prisoners at unlock on last day of the month

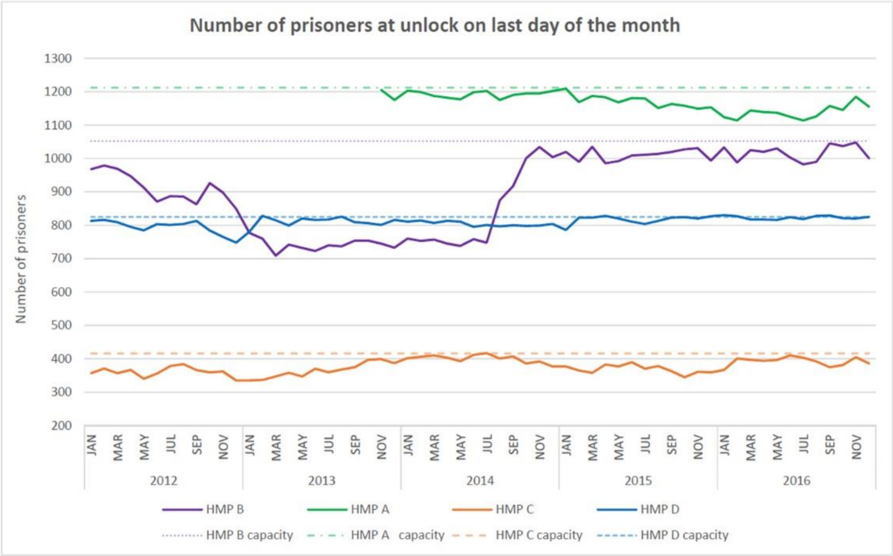

7

Appendix G: Table six standardised ACCT process costs

ACCT task per person	Initial ACCT opening and assignment of case manager by safer custody administration staff (minutes) £	Initial assessment by Case Manager (minutes) £	Case review attendance by two operational staff, one healthcare/other agency e.g., chaplaincy (minutes) £	Observation and case note entry into the ACCT documentation by Case manager (minutes) £	Post closure review (7 days after an ACCT has been shut). Interview between patient and Case Manager (minutes) £	Audit checks and data entry on the ACCT documentation once the ACCT shut by safer custody administrator (minutes) £
Time allocated	(30)	(30)	(60)	(5)	(30)	(30)
Standardised cost	4.60	6.50	39	1.05	6.50	4.60

Perry A, *et al. BMJ Open* 2019; 9:e026095. doi: 10.1136/bmjopen-2018-026095

Appendix H: Table seven training and implementation costs

Prison	Training Period	Number of staff trained	Number of training sessions	Cost for staff attending the training sessions (£)	Average cost per training sessions (£)]	Number of prisoners receiving the intervention	Overall intervention time (minutes)	Average intervention time spent per person (minutes)	Cost of intervention per head (£)	Cost of training and intervention delivery (£)	Overall cost per prisoner (£)]
HMP A	15.2.15-7.7.15	175	24	2625	172.87	26	1055	40.5	£35.17	6478	249.17
HMP B	15.2.15-7.7.15	175	24	2625	172.87	26	1055	40.5	£35.17	6478	249.17
HMP C	11.12.15-26.2.16	18	2	270	207.6	-	-	-	-	415.20	0
HMP D	23.6.16-9.8.16	8	2	120	132.6	4	90	22.5	£28.87	500.7	125.17

Perry A, *et al. BMJ Open* 2019; 9:e026095. doi: 10.1136/bmjopen-2018-026095

Appendix I: Figure 2 prisoner ACCTs, intervention and post assessment relative to baseline assessment

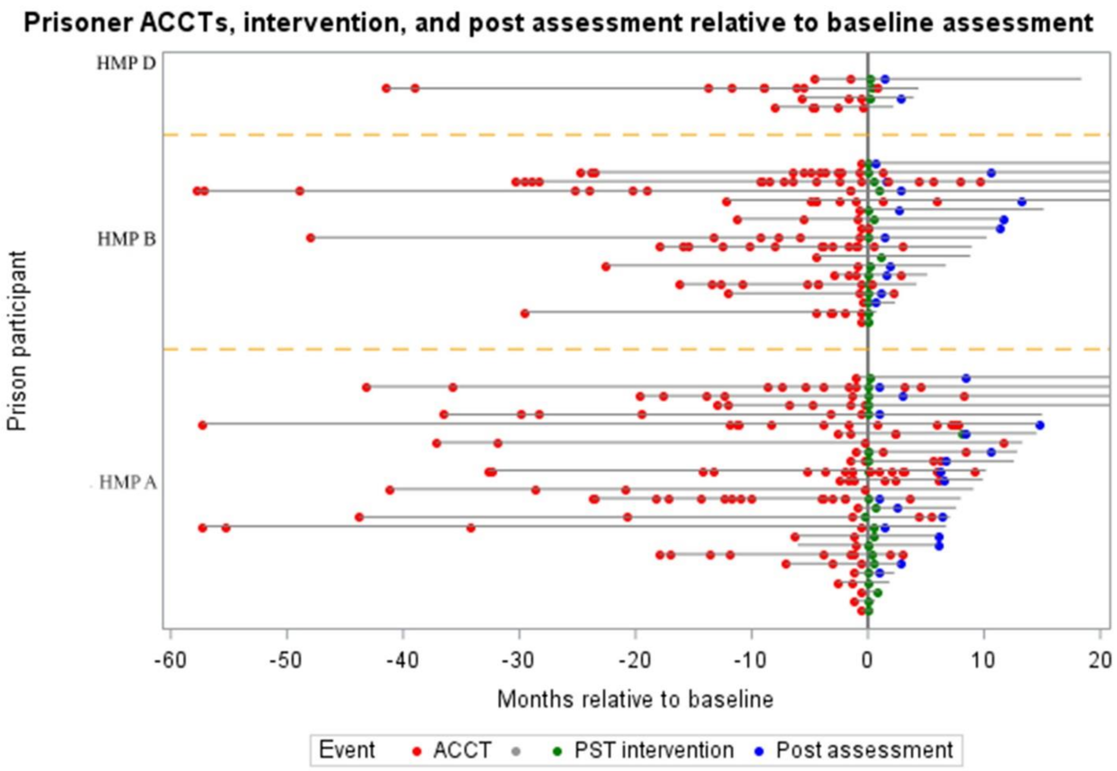

Note that ACCT data were available up to 5 months in HMP D, 12 months in HMP B, and 13 months in HMP A.

Appendix J: Figure 3 Phq-9 score at baseline and follow-up

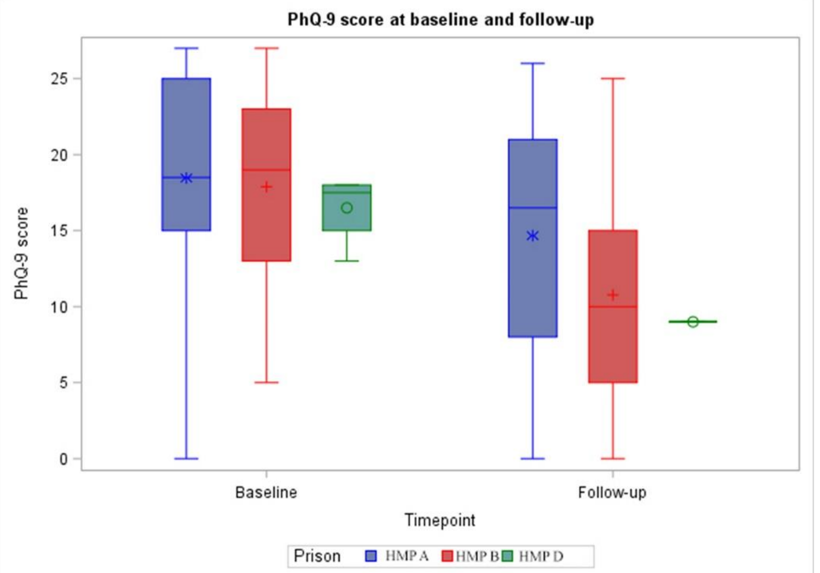

Note: follow-up took place a median of 2.8 months post-baseline, between 3 weeks and 15 months.

Perry A, *et al. BMJ Open* 2019; 9:e026095. doi: 10.1136/bmjopen-2018-026095
