# Supplementary material for: Problem-solving training: assessing the feasibility and acceptability of delivering and evaluating a problem-solving training model for front-line prison staff and prisoners who self-harm
Source: BMJ Open. 2019 Oct 3;9(10):e026095. doi: 10.1136/bmjopen-2018-026095 (PMC6797432; doi:10.1136/bmjopen-2018-026095)
Supplement: Author's manuscript [file bmjopen-2018-026095.draft_revisions.pdf]

BMJ Open is committed to open peer review. As part of this commitment we make the peer review history of every article we publish publicly available.

When an article is published we post the peer reviewers' comments and the authors' responses online. We also post the versions of the paper that were used during peer review. These are the versions that the peer review comments apply to.

The versions of the paper that follow are the versions that were submitted during the peer review process. They are not the versions of record or the final published versions. They should not be cited or distributed as the published version of this manuscript.

BMJ Open is an open access journal and the full, final, typeset and author-corrected version of record of the manuscript is available on our site with no access controls, subscription charges or pay-per-view fees (http://bmjopen.bmj.com).

If you have any questions on BMJ Open's open peer review process please email info.bmjopen@bmj.com

BMJ Open

Problem-Solving Training: assessing the feasibility and acceptability of delivering and evaluating a problem-solving training model for front-line prison staff and prisoners who self-harm.

Journal:	*BMJ Open*
Manuscript ID	bmjopen-2018-026095
Article Type:	Research
Date Submitted by the Author:	03-Sep-2018

Complete List of Authors:	perry, Amanda; Unviersity of York, Health Sciences; University of York<br>Waterman, Mitchell; University of Leeds, Faculty of Medicine and Health<br>House, Allan; Academic Unit of Psychiatry,<br>Wright-Hughes, Alexandra; University of Leeds, Clinical Trials Research Unit<br>Greenhalgh, Joanne; University of Leeds, Sociology and Social Policy<br>Farrin, Amanda; University of Leeds, Clinical Trials Research Unit<br>Richardson, Gerry; University of York, Centre for Health Economics<br>Hopton, Ann; Universiy of York, Health Sciences<br>Wright, Nat; Spectrum Community Health CIC

Keywords:	Prisoners, Suicide & self-harm < PSYCHIATRY, Feasibility, EDUCATION & TRAINING (see Medical Education & Training)

SCHOLARONE™
Manuscripts

For peer review only

Title: Problem-Solving Training: assessing the feasibility and acceptability of delivering and evaluating a problem-solving training model for front-line prison staff and prisoners who self-harm.

AUTHORS:

Amanda E. Perry[1]

Mitch G. Waterman[2]

Allan O. House[3]

Alexandra Wright-Hughes[4]

Joanne Greenhalgh[5]

Amanda Farrin[4]

Gerry Richardson[6]

Ann Hopton[1]

Nat Wright [7]

Corresponding authors email address: amanda.perry@york.ac.uk

For peer review only

[1] Department of Health Sciences, University of York
[2] Faculty of Medicine and Health, University of Leeds, Leeds LS2 9JT, UK
[3] Leeds Institute of Health Sciences, University of Leeds, Leeds LS2 9JT, UK
[4] Clinical Trials Research Unit, Leeds Institute of Clinical Trials Research, University of Leeds, Leeds LS2 9JT, UK
[5] Sociology and Social Policy, Faculty of Education and Social Science and Law, University of Leeds, LS2 9JT, UK.
[6] Centre for Health Economics, University of York, York, YO10 5DD Sociology and Social Policy, Faculty of Education and Social Science and Law, University of Leeds, LS2 9JT, UK.
7. Spectrum Community Health CIC, Wakefield, WF1 5RH.

For peer review only - http://bmjopen.bmj.com/site/about/guidelines.xhtml

ABSTRACT

Objectives: Problem-solving skills training is adaptable, inexpensive and simple to deliver. However, its application with prisoners who self-harm is unknown. The study assessed the feasibility and acceptability of a Problem-Solving Training (PST) intervention for prison staff and prisoners who self-harm, to inform the design of a large-scale study.

Design and setting: A mixed methods design used routinely collected data, individual outcome measures, an economic protocol and qualitative interviews at four prisons in Yorkshire and Humber, UK.

Participants: (i) front-line prison staff, (ii) male and female prisoners with an episode of self-harm in the previous two weeks.

Intervention: The intervention comprised a one-hour staff training session and a 30-minute prisoner session using adapted workbooks and case studies.

Outcomes: We assessed the study processes - coverage of training; recruitment and retention rates and adequacy of intervention delivery - and available data (completeness of outcome data, integrity of routinely collected data and access to NHS resource information). Prisoner outcomes assessed incidence of self-harm, quality of life and depression at baseline and at follow-up. Qualitative findings are presented elsewhere.

Results: Recruitment was higher than anticipated for staff n=280, but lower for prisoners, n=48. Retention was good with 43/48 (89%) prisoners completing the intervention, at follow-up we collected individual outcome data for 34/48 (71%) of prisoners. Access to routinely collected data was inconsistent. Prisoners were frequent users of NHS healthcare. The additional cost of training and intervention delivery was deemed minimal in comparison to ‘treatment as usual’. Outcome measures of self-harm, quality of life and depression were found to be acceptable.

Conclusions: The intervention proved feasible to adapt. Staff training was delivered but on the whole it was not deemed feasible for staff to deliver the intervention. A large-scale study is warranted, but modifications to the implementation of the intervention are required.

STRENGTHS AND LIMITATIONS OF THIS STUDY

Prison staff and prisoners were involved in the development of our questionnaires, the intervention adaptation and production of the workbooks.

The feasibility study was conducted across four prison sites including male and female prisoners.

Outcome data were collected via a variety of different sources demonstrating variability and differences in data collection procedures.

It was not deemed feasible for staff to deliver the intervention.

For peer review only

INTRODUCTION

Problem-solving skills training delivered in a systematic manner provide a non-specialist intervention that is accessible to anyone following brief training. Deficits in problem-solving skills are often found in people who self-harm and can result in reliance on others, leading to a passive as opposed to an active problem-solving approach [1-3]. Trials of problem-solving skills in the *community* demonstrate that teaching people to use brief problem-solving skills can reduce repetition of self-harm behaviour [4-6].

In prison, despite growing numbers of those who self-harm there is a lack of psychological support for prisoners and a recognised need to provide adequate staff training (NICE Guidance CG133: https://www.nice.org.uk/guidance/cg133/chapter/2-Research-recommendations). Evaluations of trials in prisons have explored alternative therapy models for those who self-harm (e.g., cognitive behaviour therapy and interpersonal psychotherapy), but such interventions require the use of extensive resources, large numbers of therapy sessions and qualified clinical therapists, making them inaccessible for prisoners who might only be incarcerated for short periods of time [7, 8].

Use of a brief PST intervention offers one solution to this problem. It has the advantages of being deliverable by any member of staff making it an attractive, inexpensive opportunity to reduce repeat self-harm. However, it is unclear whether the training is acceptable, or whether it can be implemented by staff in this setting. We therefore assessed the feasibility and acceptability of adapting an existing PST for frontline prison staff with the intention that they would deliver the intervention to prisoners who self-harm. This article reports on the acceptability and feasibility of the training, and the implementation of the intervention. Detailed methods on the qualitative findings are submitted elsewhere [9].

MATERIALS AND METHODS

Study design and setting

The study used a mixed-methods design - including quantitative collection of routine data, individual outcome measures and economic resource data, and information from staff to identify how much time was spent on 'usual care'.

The study took place in four prisons in Yorkshire and Humber between September 2014 and May 2017. The study sites included two male adult local prisons where the majority of prisoners are awaiting sentence (housing up to 1,212 and 1052 prisoners, prisons A and B), one female prison (housing up to 416: prison C) and one resettlement prison where sentenced prisoners are housed prior to transfer (housing up to 825: prison D).We report on our intervention using the template for intervention description and replication (TIDier) checklist [10].

Patient and Public Involvement

Our research questions and outcome measures were not informed by prisoner preferences and prisoners were not involved in the recruitment to the study. Prisoners did contribute significantly to the format and adaptation of the training materials. The training materials were printed from within the prison by prisoners. The results were disseminated using an A4 summary sheet which was sent to prisoners and prison staff.

The intervention

For peer review only - http://bmjopen.bmj.com/site/about/guidelines.xhtml

The PST intervention was originally devised in New Zealand for patients who self-harm in the community [11]. The seven-step model includes 'getting the right attitude' (step one), reflection and recognising triggers (step two), defining a clear problem (step three), brain storming solutions (step four), decision making (step five), making a plan (step six) and reviewing progress (step seven). Problem-solving skills are an approach that encourages an individual to address their problems in a proactive manner using the systematic seven-step process. The theory behind social problem-solving is well established and often forms part of more extensive cognitive behaviour therapy sessions [12, 13].

The adaptation of the training and intervention materials

The training was adapted using focus groups. They were used to ensure (i) the appropriateness and context of the case materials and (ii) to promote discussion with staff and prisoners about their views on how the study might work.

Staff training and recruitment

Staff were recruited with the help of prison liaison staff who assisted with room bookings, shift management and allocation of individuals to attend the training course. Using estimates provided by the prison about: the number, and type of staff employed by the prison, we estimated a feasible recruitment goal of 125 trained staff across the four sites in our 12-month training period.

Staff received a one-hour training session between March 2015 and August 2016. Training was delivered by the research team in a flexible manner (e.g., during induction or on a lunchtime). Eligible prison staff included anyone with responsibility for prisoners who were at risk of self-harm and who were monitored under the prison system (Assessment Care in Custody Teamwork: ACCT[14]). Invited staff groups included management, probation, teaching, prison officers, chaplaincy, psychologists, specialist suicide prevention assessors and nursing staff. All staff receiving the training gave full informed consent.

Recruitment and implementation of the intervention with prisoners who self-harmed

Recruitment of prisoners occurred at prison sites A, B and D. In site C access to the prison site was limited. Our feasible recruitment goal of 120 were based on access to three sites and monthly prison information on the numbers of those 'at risk'.

Prisoners were identified via the ACCT register and approached by a member of the research team or prison staff. Eligible prisoners were 1) >16 years or over and (2) with an episode of self-harm or attempted suicide in the previous two weeks. Prisoners were excluded if (i) an ACCT was opened for reasons other than actual self-harm or attempted suicidal behaviour, (ii) they were deemed too unwell by prison staff, or (iii) posed a risk to the researchers. Consenting participants completed baseline and follow-up questionnaires.

The entirety of the intervention was delivered in a 30-minute session. The session demonstrated use of the seven-steps using the booklets and case studies developed in the focus groups. Prisoners were invited to attend subsequent follow-up sessions to assess progress and support their engagement with the intervention.

Feasibility and acceptability measures

For peer review only - http://bmjopen.bmj.com/site/about/guidelines.xhtml

Data were collected on rates of recruitment, consent and retention for staff and prisoners. Reasons for non-participation and withdrawal were collected, where possible.

For outcome measures we assessed feasibility and acceptability by recording completion and follow-up rates. Typically, completion rates <50% are taken to indicate non-feasibility, >75% as indicating feasibility, and 51-74% as ambiguous - requiring modifications to design or implementation plans and reconsideration.

Our primary outcome proposed for a definitive trial was incidence of self-harm. Data on self-harm and/or attempted suicides were recorded at three months prior to baseline, baseline, post-intervention and at three months follow-up (or up to point of release or transfer) from SystmOne using the search terms 'self-harm' and 'F213'[7].

Individual secondary outcomes at baseline and follow-up included measurement of quality of life using the EQ-VAS: [15] and depression using the PHQ-9:[16]. The EQ-VAS is a self-rated questionnaire providing description of the subject's current health in five dimensions i.e., mobility, self-care, usual activities, pain/discomfort and anxiety/depression and is rated into one of three degrees of disability (severe, moderate or none). The PHQ-9 is a well validated tool for the measurement of depression with robust psychometric properties, reliability and validity in adult community populations.

Costs were estimated by: (i) completion of a self-report questionnaire reporting on access to NHS treatment before, during and after the study (ii) staff interviews to ascertain the average time spent on each ACCT process, (iii) a case note review of eleven prisoner ACCT documents to record the amount of staff time involved in the ACCT procedure, and (iv) the number of training sessions, numbers of staff attending each session, and the duration and timing of each training session.

We obtained routinely collected electronic ACCT data consisting of individual and monthly ACCT information between January 2012 and December 2016. The time period of the data collection was prescribed by the individual prison data collection protocols (table 1 supplementary materials). We found that data were comparable from our four prison sites across this time period. Prior to 2012 the comparability of data and access to data were found to be limited and December 2016 was the latest date for which all prisons had complete data.

DATA ANALYSIS

Data were summarised, by prison, using descriptive summary statistics. The information included the description of the focus group participants, the number of training sessions and staff attending training sessions. The feasibility and success of recruitment of prisoners to the study is evaluated through summaries of the screening, eligibility, consent and recruitment processes.

A summary of the variability of available routine data across: outcomes, prison and wings (where available), and the estimated cost of usual care were informed using staff information and case review process. Delivery and implementation of the PST intervention were estimated using the numbers of training sessions, numbers of staff attending, standardised staff costs, facilitator time in the delivery of the session and preparation for each session alongside the cost of materials. Summary statistics for prisoner's baseline characteristics and

[7] F213 is the title of the form used by the prison service to record incidents of self-harm behaviour

For peer review only - http://bmjopen.bmj.com/site/about/guidelines.xhtml

outcomes for the incidence of self-harm behaviour, quality of life, depression and information on access to NHS treatment were recorded.

RESULTS

Feasibility assessment

Adapting and developing the materials

During 2015 staff and prisoners were nominated by each prison to participate in focus groups. 31 staff participants attended (table two supplementary materials). They comprised of mainly operational 17 /31(55%) or managerial 6/31 (19%) staff with a mean age of 37 years. The majority were female 20/31 (66%), spoke English as their first language 27/31 (88%) and were British 27/31 (90%).

Six focus groups involving 67 prisoners, included mainly male prisoners 56/67 (83.6%) with a mean age of 39.8 years (SD 9.63). There were fewer prisoners on remand or first-time offenders involved in the focus groups, compared to recruited prisoners for the study (table three supplementary materials). The process resulted in two gender-specific picture booklets and a series of exercises with associated case study scenarios that were used in the training and delivery of the intervention.

Coverage of staff training and recruitment

280 prison staff were trained between March 2015 and August 2016 (see figure 1). Training was delivered by the research team to staff groups with a mean size of 8 staff (range of group sizes 2-19). Recruitment of staff to training sessions appeared to be acceptable and feasible.

[Insert Figure 1 here]

Staff trained were mainly operational (120, 43%) or healthcare staff (78, 28%); other staff included a number of voluntary, managerial, admin, education, and offender manager probation staff. Mean age of staff trained was 42 years, 59% were male, and almost all spoke English as their first language and were British. Trained staff had spent a median of 8 years (range <1 month – 36 years) working in the prison service (see table 1).

[Insert table 1 here]

Screening and recruitment of prisoners

During the three-month recruitment period at each site a total of 281 prisoners were eligible to participate as per the study criteria. Of these, 106/281 (37%) were released or transferred to another prison site prior to invitation to attend an appointment in healthcare. The average time between identification of an eligible participant and meeting them to inform them about the studied varied between at each site between one and three weeks.

Of the remaining 175 (62%), 95/175 were not seen in healthcare for a variety of reasons. These included: 66/95 (69%) people who did not attend their appointment to be informed about the study following three consecutive invitations, 9/95 (9%) were considered too dangerous to approach, 6/95 (6%), lacked sufficient capacity, 5/95 (5%), were transferred or released prior to attending the appointment, 8/95 (8%), were not approached by the research team due to limited resources within the team and one person died 1/95 (1%). Of the remaining 83 people, 6 (7%) attending the appointment were deemed not eligible reporting

For peer review only - http://bmjopen.bmj.com/site/about/guidelines.xhtml

no incident of actual self-harm behaviour. For the remaining 75 people 29/75 (39%) did not consent to take part leaving 48/75 (66%) consenting participants.

[Insert Figure 2 here]

The median age of prisoners was 30 years (range 59 to 58 years). All but three were White British, and all spoke English as their first language. Two thirds 32/48 (67%) were single and had never married; the majority smoked 39/48 (83%) and did not have a physical or learning disability (36/48 77% and 33/48 69%). Only a minority of prisoners recruited from prison B and none of those in prison D were on remand, whilst almost half of prison A recruited prisoners were on remand 22/48 (46%). Only a quarter were first time offenders 12/48 (25%), the number of times prisoners had been in prison ranged up to 50, with a median of 3 times. The median length of sentence was 27 months, with prisoners having spent a median of 3 months (range 2 days to 2 years) in their current and a median of 9 months left in prison (range 3 days to 15 years). For self-harm details see table four supplementary materials.

Retention

5/48 (10%) participants did not complete the intervention and withdrew from the study (figure 2); although general reasons were not provided for withdrawal. We tracked the transfer of 7/48 (15%) prisoners between our study sites. Transfer reasons included the progression of prisoners through their sentence (e.g., from a local prison to our resettlement prison) or were unexpected due to a security breech.

Adequacy of intervention delivery with prisoners who self-harmed

Between August 2015 and June 2016 delivery of the intervention by staff occurred for only two prisoners. At prison C the research team had limited access to deliver the intervention and instead the prison decided to take the booklets and distribute them on the wings to target bullying. For the remaining 46/48 (96%) participants the intervention was delivered by members of the research team in the healthcare unit.

The median time spent on intervention delivery was 40 minutes per prisoner, (range 30-90 minutes). The overall time spent with the researcher, including the baseline assessment, intervention delivery, follow up questionnaire for outcomes and qualitative interview averaged a median of 80 minutes, (range 30 minutes up to 2 hours 30 minutes) over a period of 1-7 contact appointments. In interviews, the intervention was acceptable to prisoners who received the intervention [5].

Acceptability of outcome measures

Use of routinely collected data to inform large-scale study

We found that reporting of self-harm data was complicated and recorded by several different methods, with variability in recording and differing definitions of self-harm across the four sites (table five supplementary materials). Figure 3 shows the variability in monthly number of ACCTs opened at each site per 100 prisoners. The greatest variability of open ACCTs was displayed in prison C (our female site): figures 1 and 2 supplementary materials provide further details.

[Insert Figure 3 here]

Estimating the costs

For peer review only - http://bmjopen.bmj.com/site/about/guidelines.xhtml

The estimated cost of usual care were gathered in staff interviews whereby staff told us how much time on average they spent conducting each element of the ACCT process (figure 3 supplementary materials). Using this data each task in the ACCT process was assigned a proportionate salary costs (table six supplementary materials).

The eleven case reviews identified a total of twenty-four ACCTS documents. For two prisoners the ACCT was in use at the point of data collection providing a conservative estimate of cost. The numbers of case reviews for each prisoner ranged from one to thirty-three, the number of staff observations ranged between 0 and 5520. The total administrative costs for the eleven prisoners was estimated at £21,650, an average of £1,968 per prisoner (range £375-£6416).

Training costs included a notional hourly rate (of £15 per person) to release staff attending the training session. The delivery costs included travel, preparation time and cost of course materials. Across sites we estimated training and intervention costs of between £500 and £6406 equating to a cost per prisoner of between £125 and £246 (table seven supplementary material). Overall it proved feasible to gather resource information to provide a cost estimate of usual care, delivery of training and implementation of the intervention.

Prisoner outcomes

100% of those agreeing to participate in the study completed the baseline assessment. Follow-up times varied considerably, taking place a median of 2.8 months after recruitment but up to a maximum of 15 months for one prisoner (see figure 4). The timing of follow up assessments fell into three clusters, the largest cluster taking place within the first three months post recruitment, a further set taking place between 6 and 8 months post recruitment in prison A. Follow-up was affected when access to prison A was halted for a three-month period. Overall the average follow-up rate for questionnaire returns was 34/48 (71%) across the three sites. The changes in scores reflect them as potentially useful outcome measures that could be used in a large-scale evaluation.

[Insert Figure 4 here].

Primary outcome: incidence of self-harm behaviour

Incidence of self-harm behaviour appeared to decrease over the life time of the project. At 3 months prior to baseline, 32/48 (66%) prisoners had harmed themselves. This reduced to 9/48 (18%) prisoners at post-test. Data on prisoner ACCTs are shown in figure three supplementary materials.

Secondary outcomes

Quality of life

A total of 32/48 (66%) of individuals completed full information on the EQ-VAS. The baseline mean score (0.504, SD 0.34) fell post intervention (0.625, SD, 0.347).

Depression

At baseline, median scores were high at 18 and most prisoners had either moderately severe 18/48 (38%) or severe depression 20/48 (40%). Prisoners' at follow-up had lower depression

For peer review only - http://bmjopen.bmj.com/site/about/guidelines.xhtml

scores with just 7/48 (15%) classed as moderately severely depressed, and 13/48 (27%) as severely depressed.

Access to NHS services

All 48 prisoners had received some NHS service provision whilst in prison. Access to a GP (range 1-10 appointments), pharmacist (daily drug dispensing) or duty nurse (range 1-35 appointments) appointments were the most cited points of contact. 35/48 (73%) prisoners reported accessing mental health services, two reported access to a psychological therapy. Just under a quarter 11/48 (23%) had experienced a hospital admission and 13/48 (27%) (range 1-9) reported attending accident and emergency in the three months prior to incarceration. In all cases, admissions were related to synthetic cannabinoids intake, overdose, attempted hanging or feeling suicidal. There were no adverse events reported as part of the PST intervention during the study period.

DISCUSSION

The study aimed to assess the feasibility and acceptability of adapting and implementing a brief PST intervention for prison staff and prisoners at risk of self-harm. Our results indicate that staff can be trained in using these skills though most were unable to implement them with those who self-harmed. Prison staff faced severe time pressures, and limited resources making it difficult to accommodate the translation of knowledge into practice. This is a common problem in the design and implementation of complex interventions in organisations other than healthcare [17]. These findings emerged during the implementation phase. The brief nature of the training sessions themselves did not, perhaps, facilitate the expression of these doubts or tackle approaches to translation of skills into practice in a pressured environment.

Attrition from the study sample by prisoners was minimal due to the 30-minute intervention design. Previous prison trials have demonstrated relatively high levels of attrition. In our study (despite a lack of access to one site for three months) we managed to produce encouraging follow-up rates (71%) suggesting that our outcomes were acceptable. Our findings are comparable with other pilot trials of self-harm in prisons [7, 8] and trials of suicide prevention more broadly in the community [18]. We were able to track participants through our sites. This allowed us to collect follow-up data on seven participants who were released from prison A, and either returned back to the same prison during the study period or were transferred to prison C prior to release. Prison function is therefore an important consideration. Turnover of prisoners at our local prison sites (e.g., prisons A and B) was considerably greater than in our resettlement prison. This finding is supported elsewhere with data provided from prison A in a recent Inspectorate report showing that 430/1109 (38%) were imprisoned for less than three months in 2017. Prisoners followed from prison A through to prison C were notably in a better position to engage with training when in the resettlement prison. This system of 'tracking' participants provides a potential mechanism to ensure adequate follow-up in a large-scale study.

There were limitations with the development of our economic protocol in the assumptions made with regards to costs for usual care which are not necessarily representative. Access, quality and consistency of these data varied across the prison sites and led us to conclude that such routine data could only be used to measure the impact of any future evaluation if additional data were provided or stricter collection protocols and monitoring were deployed.

For peer review only - http://bmjopen.bmj.com/site/about/guidelines.xhtml

We also propose that any new study should include individual self-report information and information from local and national data sources. This method is not dissimilar to other data collection mechanisms in two pilot trials of self-harm in UK prisons where prisoners report suicidal behaviours, thoughts and feelings [7, 19].

In designing a large-scale study, we have sufficient information to inform our outcomes of measurement and feasibility of data collection. However, alternative implementation mechanisms need to be identified prior to any large-scale study. Our qualitative findings (submitted elsewhere) suggest two alternative options: first, use of trusted prisoners as 'problem-solving champions' to deliver the skills to peers on the wings and/or second, delivery of problem-solving skills to prisoners through education classes.

CONCLUSIONS

The study suggests that the modified version of PST, adapted for training, was acceptable to prisoners. Although the study demonstrated that it was currently not feasible to deliver the intervention using prison staff it provides insight into how such an intervention with prisoner-staff involvement can be adapted for use in a different environment.

Author Affiliations:

Department of Health Sciences, University of York
Faculty of Medicine and Health, University of Leeds, Leeds LS2 9JT, UK
Leeds Institute of Health Sciences, University of Leeds, Leeds LS2 9JT, UK
Clinical Trials Research Unit, Leeds Institute of Clinical Trials Research, University of Leeds, Leeds LS2 9JT, UK
Centre for Health Economics, University of York, York, YO10 5DD
Spectrum Community Health CIC, Wakefield, WF1 5RH.

Acknowledgements:

The authors are grateful to Spectrum Community Health CIC and previously Leeds Community HealthCare Trust who agreed to host this study. To Christine Butt who helped with the collection of data at one prison site. We would also like to acknowledge the contributions of all the prison staff, prisoners, for support from the North East and Yorkshire Area Safer Custody Team and prison Governors for their willingness to take part and support this study.

Contributors:

AP, AH AND MW designed and conducted most of the study with considerable input from AH. AWH and AF took the lead in performing the statistical analyses together and JG was the lead for analysing the qualitative interviews with AP. GR and NW led the development of the economic protocol and information on the study costs. NW supported access to the prison sites and all authors provided input into the writing of the manuscript.

Funding: This paper presents independent research funded by the NIHR under its Research for Patient Benefit (RfPB) Programme (Grant Reference Number PB-PG-0211-24122).

For peer review only - http://bmjopen.bmj.com/site/about/guidelines.xhtml

Disclaimer: The views expressed are those of the author(s) and not necessarily those of the NHS, the NIHR or the Department of Health

Patient consent: Obtained

Competing interests None declared

Ethics approval: Ethical approval for the study was obtained for phase one from NHS REC approval [NRES, North East York, 28.10.14] and NOMS [1.9.14] and phases two-five [Bristol REC Centre, London South East, 6.1.15] from NHS REC approval, NOMS [6.3.15] and the Department of Health Sciences at the University of York for all phases [11.12.14]. As the material was adapted and developed for appropriate use within each prison we were granted one substantive amendment to the project from all parties during July 2015.

Provenance and peer review: Not commissioned; externally peer reviewed.

Data sharing statement: Participant level data, the full data set and statistical codes are available from the corresponding author.

Figure Legends:

Figure 1: Staff trained and participating focus groups

Figure 2:Flow of study participants through study

Figure 3: Monthly numbers of ACCTs opened per 100 prisoners

Figure 4: Time between recruitment and questionnaire follow-up assessment

References

1. Linehan, M.M., et al., *Interpersonal Problem-Solving and Parasuicide.* Cognitive Therapy and Research, 1987. 11(1): p. 1-12.
2. McLeavey, B.C., et al., *Interpersonal problem-solving skills training in the treatment of self-poisoning patients.* Suicide Life Threat Behav, 1994. 24(4): p. 382-94.
3. Pollock, L. and J. Williams, *Effective problem solving in suicide attempters depends on specific autobiographical recall.* Suicide and Life Threatening Behaviour,, 2011. 31: p. 386-396.
4. Hawton, K., et al., *Evaluation of out-patient counselling compared with general practitioner care following overdoses.* Psychol Med, 1987. 17(3): p. 751-61.
5. Perry, A., M. Waterman, and A. House, *Problem-Solving Training for Suicidal Prisoners*, in *The Prevention of Suicide in Prison: Cognitive Behavioural Approaches*, P. D, Editor. 2015, Taylor & Francis Ltd: UK. p. 69-83.
6. Hawton, K., et al., *Psychosocial interventions for self-harming adults.* 2016, DOI: 10.1002/14651858.CD012189.: Cochrane Database of Systematic Reviews 2016, Issue 5. Art. No.: CD012189.
7. Walker, T., et al., *The WORSHIP II study: a pilot of psychodynamic interpersonal therapy with women offenders who self-harm.* The Journal of Forensic Psychiatry & Psychology, 2017: p. 1-14.
8. Pratt, D., et al., *Cognitive Behavioural Suicide Prevention for Male Prisoners: Apilot randomised controlled trial.* Psychological Medicine, 2015. 45(16): p. 3441-3451.

For peer review only - http://bmjopen.bmj.com/site/about/guidelines.xhtml

9. Perry A E., Waterman, M.G., House, A.O. & Greenhalgh, J. [submitted], *A qualitative examination of problem solving skills in prison: the implementation of training and intervention delivery with frontline prison staff and prisoners who self-harm.* Health and Justice, Special Edition in Suicide and Self-Harm, 2018.
10. Hoffmann, T.C., et al., *Better reporting of interventions: template for intervention description and replication (TIDier) checklist and guide.* British Medical Journal, 2014. 348:g 1687(doi:10.1136/bmj.g1687.): p. 1-12.
11. Collinson, M., et al., *MIDSHIPS: Multicentre Intervention Designed for Self-Harm using Interpersonal Problem-Solving:protocol for a randomised controlled feasibility study.* Trials, 2014. 15(163): p. 1-7.

12. Evans, K., et al., *Manual Assisted Cognitive Behavioural Therapy in the Treatment of Recurrent Deliberate Self Harm: A Randomised Controlled Trial. .* Psychological Medicine, 1999. 29: p. 19-25.
13. D'Zurilla, T.J. and M.R. Goldfried, *Problem solving and behavior modification.* J Abnorm Psychol, 1971. 78(1): p. 107-26.
14. UK Ministry of Justice, *Management of prisoners at risk of harm to self, to others and from others (safer custody)*, P.S.I. 2011-64., Editor. 2013, http://www.justice.gov.uk/off enders/psis/prison-serviceinstructions- 2011 (accessed Nov 11, 2013).
15. Gusi, N., P.R. Olivares, and R. Rajendram, *The EQ-5D Health-Related Quality of Life Questionnaire*, in *Handbook of Disease Burdens and Quality of Life Measures*, V.R. Preedy and R.R. Watson, Editors. 2010, Springer New York: New York, NY. p. 87-99.
16. Kroenke, K., R.L. Spitzer, and J.B.W. Williams, *The PHQ-9: Validity of a Brief Depression Severity Measure.* Journal of General Internal Medicine, 2001. 16(9): p. 606-613.
17. Batalden, M., et al., *Co production of healthcare services.* BMJ Qual Saf, 2015. 0: p. 1-9.
18. Tarrier, N., K. Taylor, and P. Gooding, *Cognitive-behavioral interventions to reduce suicide behavior: a systematic review and meta-analysis.* Behav Modif, 2008. 32(1): p. 77-108.
19. Hawton K, O'Connor RC, and S. KEA., *Suicidal behaviour and self-harm. In Thapar A, Pine DS, Leckman JF, et al, eds. Rutter's Child and Adolescent Psychiatry 6th edn. Chichester: Wiley-Blackwell,* . Vol. 893-910.. 2015.

For peer review only

For peer review only - http://bmjopen.bmj.com/site/about/guidelines.xhtml

Table one: Demographic information of staff trained

	PrisonA (n=175)	PrisonB (n=79)	PrisonC (n=18)	PrisonD (n=8)	Total (n=280)
**Time working in the prison service (Years)**					
N	172	78	18	7	275
Mean (SD)	8.5 (8.93)	13.0 (9.04)	12.9 (8.45)	12.1 (9.91)	10.1 (9.16)
Median (Range)	6.0 (0.0, 36.0)	11.8 (0.1, 35.3)	12.5 (0.5, 25.0)	10.5 (1.3, 29.2)	8.0 (0.0, 36.0)
**Time working in this prison (Years)**					
N	172	78	18	7	275
Mean (SD)	6.2 (7.48)	11.2 (8.15)	7.9 (7.61)	9.0 (7.24)	7.8 (7.96)
Median (Range)	3.3 (0.0, 31.0)	10.9 (0.1, 35.3)	4.5 (0.3, 24.3)	7.9 (1.1, 20.8)	5.5 (0.0, 35.3)
**Since working here have you encountered an incident of self-harm?**					
Yes	119 (68.0%)	68 (86.1%)	18 (100.0%)	8 (100.0%)	213 (76.1%)
No	52 (29.7%)	11 (13.9%)	0 (0.0%)	0 (0.0%)	63 (22.5%)
Missing	4 (2.3%)	0 (0.0%)	0 (0.0%)	0 (0.0%)	4 (1.4%)
**Most recent self-harm incident?**					
Within the past 7 days	44 (37.0%)	28 (41.2%)	7 (38.9%)	3 (37.5%)	82 (38.5%)
Within the past month	24 (20.2%)	17 (25.0%)	4 (22.2%)	0 (0.0%)	45 (21.1%)
Two months or more	20 (16.8%)	8 (11.8%)	2 (11.1%)	1 (12.5%)	31 (14.6%)
Missing	31 (26.1%)	15 (22.1%)	5 (27.8%)	4 (50.0%)	55 (25.8%)
**Type of incident?**					
Self-poisoning	10 (8.4%)	1 (1.5%)	0 (0.0%)	1 (12.5%)	12 (5.6%)
Self-injury	94 (79.0%)	61 (89.7%)	16 (88.9%)	6 (75.0%)	177 (83.1%)
Mixed self-poisoning and self-injury	7 (5.9%)	4 (5.9%)	2 (11.1%)	1 (12.5%)	14 (6.6%)
Suicide	7 (5.9%)	2 (2.9%)	0 (0.0%)	0 (0.0%)	9 (4.2%)
Missing	1 (0.8%)	0 (0.0%)	0 (0.0%)	0 (0.0%)	1 (0.5%)
**Attended self-harm training?**					
Yes	74 (42.3%)	48 (60.8%)	13 (72.2%)	3 (37.5%)	138 (49.3%)
No	96 (54.9%)	30 (38.0%)	3 (16.7%)	5 (62.5%)	134 (47.9%)
Cannot recall	4 (2.3%)	1 (1.3%)	2 (11.1%)	0 (0.0%)	7 (2.5%)
Missing	1 (0.6%)	0 (0.0%)	0 (0.0%)	0 (0.0%)	1 (0.4%)
**Time since self-harm training?**					
N	66	45	13	3	127
Mean (SD)	30.5 (38.22)	20.4 (27.32)	41.7 (53.84)	19.0 (20.42)	27.8 (36.64)
Median (Range)	12.0 (0.0, 180.0)	12.0 (1.0, 120.0)	12.0 (1.0, 168.0)	12.0 (3.0, 42.0)	12.0 (0.0, 180.0)
**Who provided this training?**					
Prison service	59 (79.7%)	42 (87.5%)	8 (61.5%)	3 (100.0%)	112 (81.2%)
NHS	3 (4.1%)	0 (0.0%)	2 (15.4%)	0 (0.0%)	5 (3.6%)
Nurse Training	2 (2.7%)	1 (2.1%)	0 (0.0%)	0 (0.0%)	3 (2.2%)

For peer review only - http://bmjopen.bmj.com/site/about/guidelines.xhtml

	PrisonA (n=175)	PrisonB (n=79)	PrisonC (n=18)	PrisonD (n=8)	Total (n=280)
Other including University	3 (4.1%)	2 (4.2%)	1 (7.7%)	0 (0.0%)	6 (4.3%)
Missing	7 (9.5%)	3 (6.3%)	2 (15.4%)	0 (0.0%)	12 (8.7%)
**Length of training?**					
1 hour	19 (25.7%)	0 (0.0%)	1 (7.7%)	0 (0.0%)	20 (14.5%)
2 hours	9 (12.2%)	4 (8.3%)	1 (7.7%)	1 (33.3%)	15 (10.9%)
Half day	16 (21.6%)	14 (29.2%)	1 (7.7%)	1 (33.3%)	32 (23.2%)
Full day	13 (17.6%)	18 (37.5%)	7 (53.8%)	1 (33.3%)	39 (28.3%)
More than one day	7 (9.5%)	4 (8.3%)	3 (23.1%)	0 (0.0%)	14 (10.1%)
Missing	10 (13.5%)	8 (16.7%)	0 (0.0%)	0 (0.0%)	18 (13.0%)

For peer review only

Figure 1: Staff trained and participating focus groups

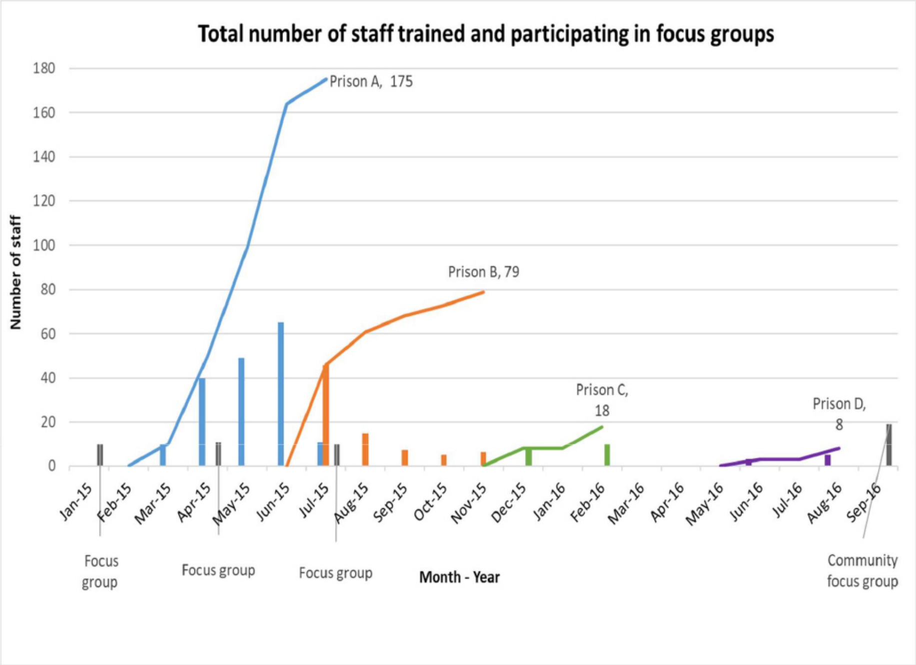

Figure 1: Staff trained and participating focus groups

90x90mm (300 x 300 DPI)

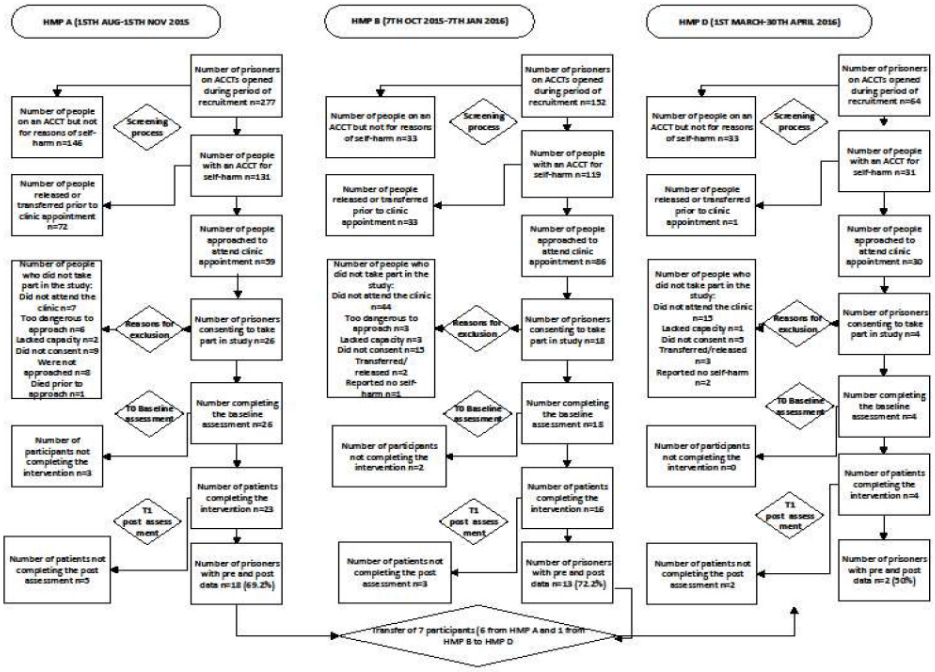

Figure 2: Flow of study participants through study

90x90mm (300 x 300 DPI)

Figure 3: Monthly numbers of ACCTs opened per 100 prisoners

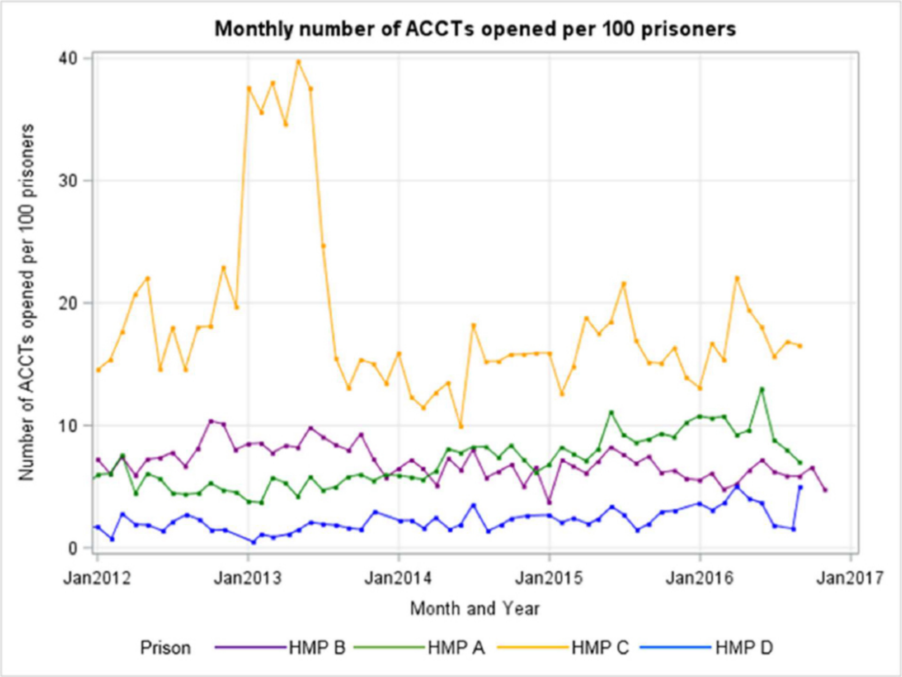

Figure 3: Monthly numbers of ACCTs openned per 100 prisoners

90x90mm (300 x 300 DPI)

Figure 4: Time between recruitment and questionnaire follow-up assessment

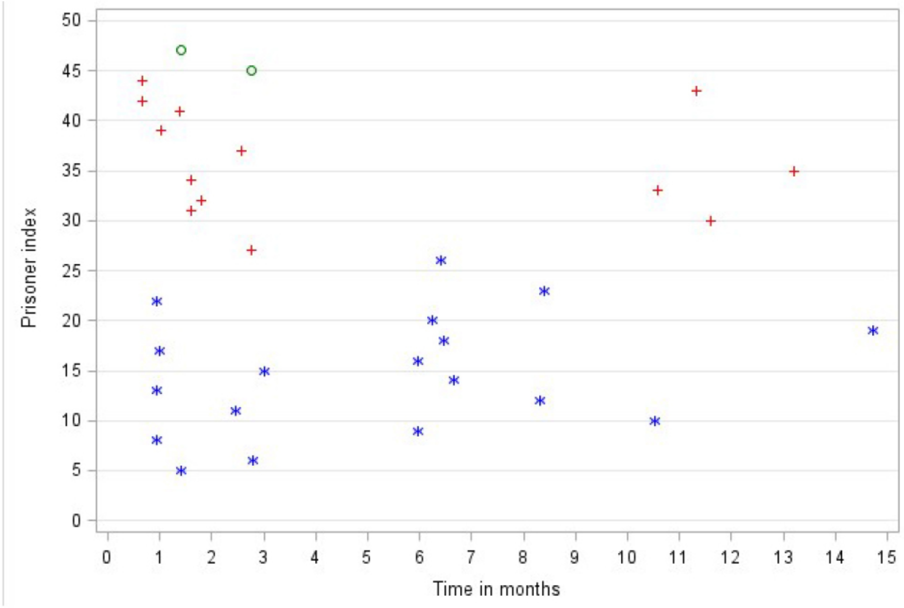

Key: * = HMP A, + = HMP B, o = HMP D.

Figure 4: Time between recruitment and questionnaire follow-up assessment

90x90mm (300 x 300 DPI)

Web Appendix Supplementary Materials

Appendix A: Table one data collection protocols

Appendix B: Table two prison staff focus group participation

Appendix C: Table three prisoner focus group participation

Appendix D: Table four prisoner self-harm details

Appendix E: Table five coding variability across prison sites

Appendix F: Figure 1 numbers of prisoners at unlock on last day of the month

Appendix G: Figure 2 monthly number of ACCTs opened per 100 prisoners

Appendix H: Figure 3 Assessment Care in Custody and Teamwork (ACCT) process

Appendix I: Table six standardised ACCT process costs

Appendix J: Table seven training and implementation costs

Appendix K: Figure three prisoner ACCTs, intervention and post assessment relative to baseline assessment

Appendix L: Figure four Phq-9 score at baseline and follow-up

For peer review only

For peer review only - http://bmjopen.bmj.com/site/about/guidelines.xhtml

Appendix A: Table one data collection protocols

Prison Site	Prison			
	**Prison A**	**Prison B**	**Prison D**	**Prison C**
**Frequency of ACCTs per year**				
2009			61	
2010			118	
2011	754		168	
2012	756	840	170	779
2013	730	734	154	718
2014	1012	645	208	688
2015	1219	798	249	729
2016 – partial*	1010	675	262	605
**Number of ACCTs per year (2012 - 2015)**				
Mean (SD)	929.3 (231.31)	754.3 (84.88)	195.3 (42.39)	728.5 (37.86)
Median (Range)	884 (730, 1219)	766 (645, 840)	189 (154, 249)	724 (688, 779)

*2016 frequencies up to September in Prison A, D and Cl, and up to November in Prison B.

For peer review only - http://bmjopen.bmj.com/site/about/guidelines.xhtml

Appendix B: Table two prison staff focus group participation

	PrisonA (n=10)	PrisonB (n=11)	PrisonC (n=10)
**Type of staff**			
Operational staff	7 (70.0%)	7 (63.6%)	3 (30.0%)
Managerial staff	0 (0.0%)	1 (9.1%)	5 (50.0%)
Healthcare staff	0 (0.0%)	1 (9.1%)	2 (20.0%)
Visitor to the prison	2 (20.0%)	0 (0.0%)	0 (0.0%)
Admin / Probation	1 (10.0%)	1 (9.1%)	0 (0.0%)
Missing	0 (0.0%)	1 (9.1%)	0 (0.0%)
**Gender**			
Male	4 (40.0%)	5 (45.5%)	3 (30.0%)
Female	6 (60.0%)	6 (54.5%)	7 (70.0%)
**Age**			
N	10	10	9
Mean (SD)	33.7 (10.07)	42.7 (11.41)	46.9 (6.15)
Median (Range)	33.0 (19, 49)	44.0 (21, 58)	47.0 (36, 56)
**First language**			
English	8 (80.0%)	11 (100.0%)	10 (100.0%)
Hungarian	0 (0.0%)	0 (0.0%)	0 (0.0%)
German	0 (0.0%)	0 (0.0%)	0 (0.0%)
Missing	2 (20.0%)	0 (0.0%)	0 (0.0%)
**Ethnic group**			
British	10 (100.0%)	11 (100.0%)	9 (90.0%)
Irish / Other white background	0 (0.0%)	0 (0.0%)	0 (0.0%)
White and Black Caribbean	0 (0.0%)	0 (0.0%)	0 (0.0%)
Indian / Pakistani	0 (0.0%)	0 (0.0%)	1 (10.0%)
**Religious preference**			
No religion	2 (20.0%)	6 (54.5%)	1 (10.0%)
Christian	8 (80.0%)	5 (45.5%)	8 (80.0%)
Muslim	0 (0.0%)	0 (0.0%)	1 (10.0%)
Hindu	0 (0.0%)	0 (0.0%)	0 (0.0%)
**Consider yourself disabled?**			
Yes	0 (0.0%)	0 (0.0%)	0 (0.0%)
No	10 (100.0%)	11 (100.0%)	10 (100.0%)
**Highest academic qualification?**			
Post Graduate	0 (0.0%)	0 (0.0%)	0 (0.0%)
Graduate	5 (50.0%)	5 (45.5%)	2 (20.0%)
A Level or equivalent	5 (50.0%)	3 (27.3%)	7 (70.0%)
GCSE or equivalent	0 (0.0%)	3 (27.3%)	1 (10.0%)

Appendix C: Table three prisoner focus group participation

	PrisonA (n=13)	PrisonB (n=43)	PrisonC (n=11)	Total (n=67)
**Are you on Remand?**				
Yes	2 (15.4%)	2 (4.7%)	1 (9.1%)	5 (7.5%)
No	11 (84.6%)	38 (88.4%)	10 (90.9%)	59 (88.1%)
Missing	0 (0.0%)	3 (7.0%)	0 (0.0%)	3 (4.5%)
**First time offender?**				
Yes	4 (30.8%)	19 (44.2%)	7 (63.6%)	30 (44.8%)
No	9 (69.2%)	23 (53.5%)	4 (36.4%)	36 (53.7%)
Missing	0 (0.0%)	1 (2.3%)	0 (0.0%)	1 (1.5%)
**Age first entered prison (years)**				
N	13	42	11	66
Mean (SD)	21.3 (7.61)	30.0 (13.13)	40.5 (12.71)	30.0 (13.35)
Median (Range)	18.0 (15.0, 40.0)	27.0 (13.0, 61.0)	39.0 (18.0, 60.0)	26.0 (13.0, 61.0)
**Number of times in prison?**				
N	13	40	11	64
Mean (SD)	7.2 (8.67)	2.8 (3.12)	1.2 (0.40)	3.4 (4.94)
Median (Range)	4.0 (0.0, 30.0)	1.5 (1.0, 15.0)	1.0 (1.0, 2.0)	1.0 (0.0, 30.0)
**Time spent in this prison? (months)**				
N	13	42	11	66
Mean (SD)	9.2 (10.66)	29.0 (25.27)	34.8 (26.98)	26.0 (24.71)
Median (Range)	6.0 (1.0, 42.0)	24.0 (2.0, 102.0)	24.0 (11.0, 84.0)	16.0 (1.0, 102.0)
**Length of sentence (months)**				
N	11	38	11	60
Mean (SD)	167.5 (344.02)	208.5 (231.48)	181.9 (108.94)	196.1 (236.56)
Median (Range)	30.0 (18, 1188)	120.0 (8, 666)	168.0 (42, 333)	126.0 (8, 1188)
**Months left until sentence expiry**				
N	9	30	8	47
Mean (SD)	36.1 (44.62)	64.0 (59.95)	76.5 (56.46)	60.8 (57.15)
Median (Range)	16.3 (3.7, 120.2)	41.0 (0.8, 257.9)	56.4 (6.2, 154.6)	40.2 (0.8, 257.9)

For peer review only - http://bmjopen.bmj.com/site/about/guidelines.xhtml

Appendix D: Table four prisoner self-harm details

	PrisonA (n=26)	PrisonB (n=18)	Prison D (n=4)	Total (n=48)
**Ever harmed yourself?**				
Yes	26 (100.0%)	18 (100.0%)	4 (100.0%)	48 (100.0%)
**Time since most recent self-harm (months)**				
N	26	16	4	46
Mean (SD)	1.0 (0.72)	0.7 (0.45)	1.5 (1.86)	1.0 (0.79)
Median (Range)	1.0 (0.0, 3.0)	0.7 (0.0, 1.5)	0.8 (0.1, 4.2)	0.7 (0.0, 4.2)
**Self-Harm frequency**				
Every day	1 (3.8%)	0 (0.0%)	0 (0.0%)	1 (2.1%)
Twice a week	3 (11.5%)	2 (11.1%)	0 (0.0%)	5 (10.4%)
Once a week	4 (15.4%)	1 (5.6%)	1 (25.0%)	6 (12.5%)
Every two weeks	0 (0.0%)	4 (22.2%)	1 (25.0%)	5 (10.4%)
Once a month	3 (11.5%)	2 (11.1%)	0 (0.0%)	5 (10.4%)
3 monthly	3 (11.5%)	1 (5.6%)	1 (25.0%)	5 (10.4%)
Less often than three monthly	12 (46.2%)	7 (38.9%)	1 (25.0%)	20 (41.7%)
Missing	0 (0.0%)	1 (5.6%)	0 (0.0%)	1 (2.1%)
**Type of most recent self-harm**				
Ligature	2 (7.7%)	3 (16.7%)	0 (0.0%)	5 (10.4%)
Cutting	14 (53.8%)	11 (61.1%)	4 (100.0%)	29 (60.4%)
OD Medication/ Recreational drug overdose	6 (23.1%)	4 (22.2%)	0 (0.0%)	10 (20.8%)
Electrocution	1 (3.8%)	0 (0.0%)	0 (0.0%)	1 (2.1%)
Hunger strike	3 (11.5%)	0 (0.0%)	0 (0.0%)	3 (6.3%)
**How easy was it for you to get help?**				
Very Easy	6 (23.1%)	4 (22.2%)	0 (0.0%)	10 (20.8%)
Took some time	10 (38.5%)	6 (33.3%)	2 (50.0%)	18 (37.5%)
There was no help available	7 (26.9%)	4 (22.2%)	0 (0.0%)	11 (22.9%)
I didn't bother to ask	3 (11.5%)	3 (16.7%)	2 (50.0%)	8 (16.7%)
can't remember	0 (0.0%)	1 (5.6%)	0 (0.0%)	1 (2.1%)

For peer review only - http://bmjopen.bmj.com/site/about/guidelines.xhtml

Appendix E: Table five coding variability across prison sites

Prison B	Prison A	Prison C	Prison D
**Prior to Nov 2013:** Not available<br>**Nov 2013 – Aug 2014:** coded as self-harm or concerns<br>**Aug 2014 – Oct 2015:** free text reasons<br>**Oct 2015 – present:**<br>1. Suicide attempt or statement of intent to take own life<br>2. Self-injury or statement to self-harm<br>3. Unusual behaviour/talk<br>4. Very low mood<br>5. Drug Alcohol Withdrawal<br>6. Other concerns<br>7. Self-harm warning received from court<br>*Not possible to distinguish acts of self-harm and suicide from intent, statements, or concerns.*	Recorded as free text.<br><br>*Not always possible to distinguish acts of self-harm and suicide from intent, statements, or concerns.*	Not available	**Prior to mid-2016:**<br>1. Suicide Attempt<br>2. Statement/thoughts of intent to kill self<br>3. Self-Harm<br>4. Statement of intent/thoughts to self-harm<br>5. Unusual Behaviour<br>6. Low mood<br>7. Problems related to Drug / Alcohol withdraw<br>8. External Concerns<br>9. Deportation<br>10. Bullying<br>11. Other<br>**Mid-2016 to present:**<br>1. Suicide attempt or Statement of intent to take own life<br>2. Self-Injury or Statement to Self-Harm<br>3. Unusual behaviour/Talk<br>4. Low Mood<br>5. Drug Alcohol Withdrawal<br>6. Other Concerns<br>*Method coding also provided and varies*
**Following coding:**<br>• 39% Related to SH or suicide<br>• 20% Other<br>• 41% Missing	**Following coding:**<br>• 36% due to SH incident or suicide attempt<br>• 39% related to SH or suicide<br>• 25% Other<br>• <1% Missing		**Following coding:**<br>• 39% due to a SH incident or suicide attempt<br>• 24% related to SH or suicide<br>• 33% Other<br>• 4% Missing

For peer review only

For peer review only - http://bmjopen.bmj.com/site/about/guidelines.xhtml

Appendix F: Figure 1 numbers of prisoners at unlock on last day of the month

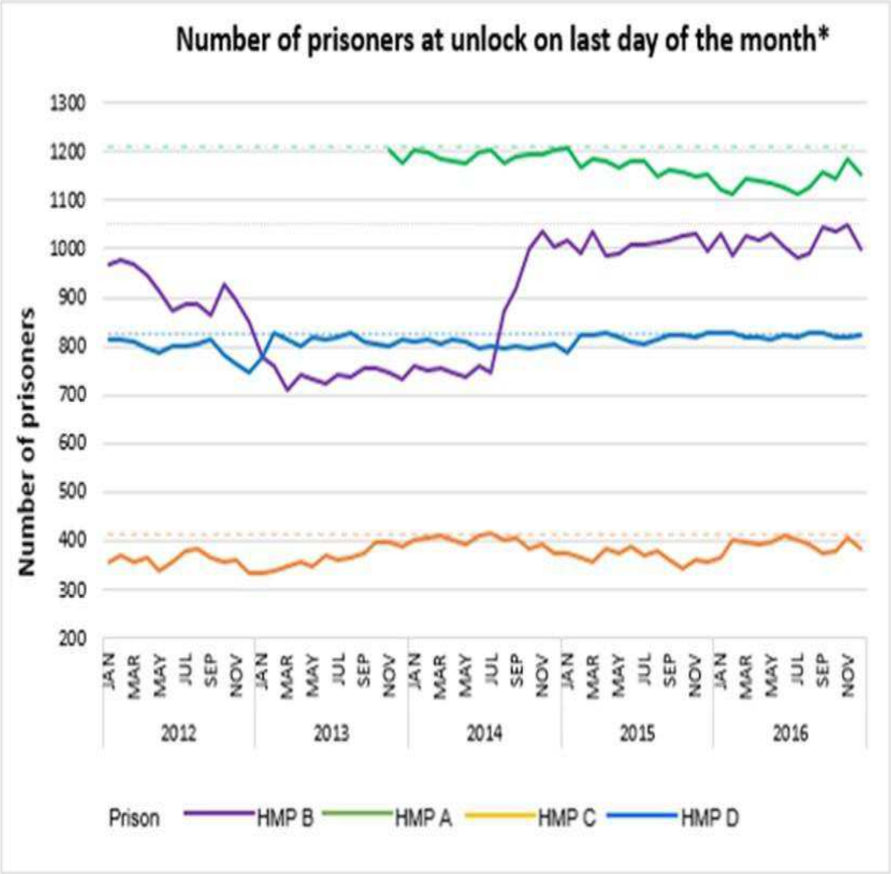

*---- indicates capacity

Appendix G: Figure 2 monthly number of ACCTs opened per 100 prisoners

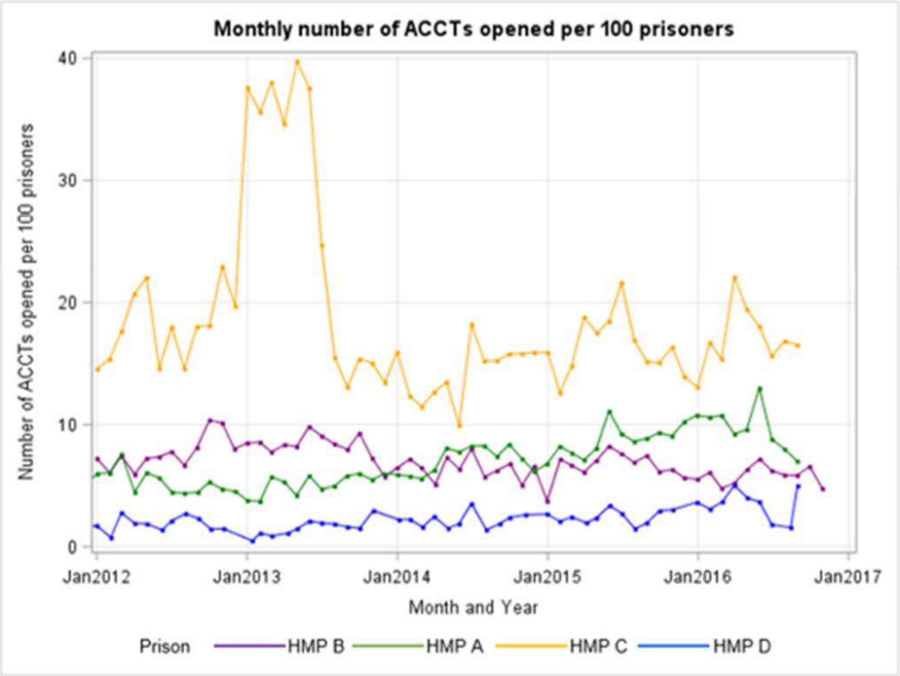

For peer review only - http://bmjopen.bmj.com/site/about/guidelines.xhtml

Appendix H: Figure 3 Assessment Care in Custody and Teamwork (ACCT) process

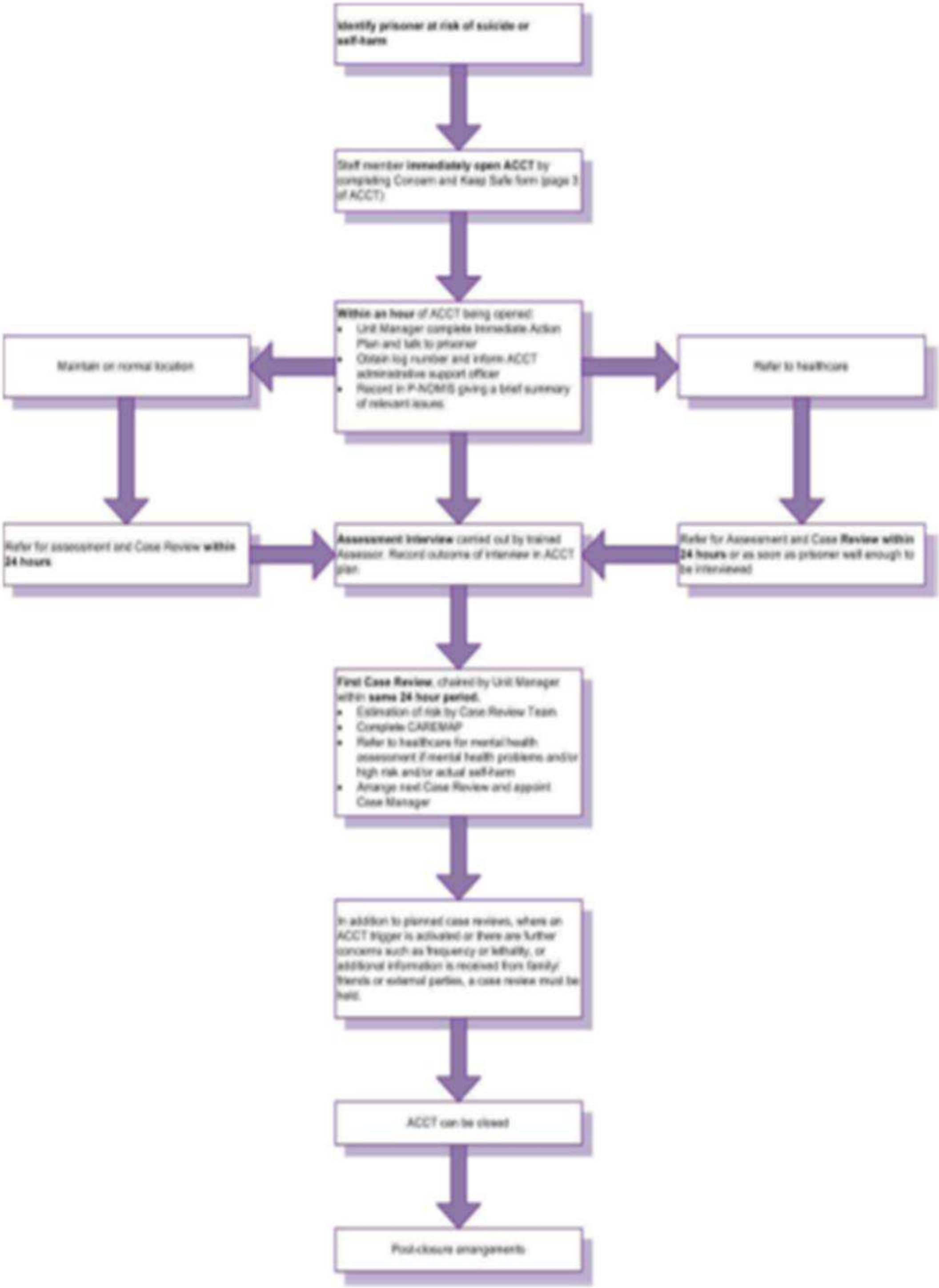

Appendix I: Table six standardised ACCT process costs

ACCT task per person	Initial ACCT opening and assignment of case manager by safer custody administration staff (minutes) £	Initial assessment by Case Manager (minutes) £	Case review attendance by two operational staff, one healthcare/other agency e.g., chaplaincy (minutes) £	Observation and case note entry into the ACCT documentation by Case manager (minutes) £	Post closure review (7 days after an ACCT has been shut). Interview between patient and Case Manager (minutes) £	Audit checks and data entry on the ACCT documentation once the ACCT shut by safer custody administrator (minutes) £
Time allocated	(30)	(30)	(60)	(5)	(30)	(30)
Standardised cost	4.60	6.50	39	1.05	6.50	4.60

For peer review only - http://bmjopen.bmj.com/site/about/guidelines.xhtml

Appendix J: Table seven training and implementation costs

Prison	Training Period	Number of staff trained	Number of training sessions	Cost for staff attending the training sessions (£)	Average cost per training sessions (£)]	Number of prisoners receiving the intervention	Overall intervention time (minutes)	Average intervention time spent per person (minutes)	Cost of intervention per head (£)	Cost of training and intervention delivery (£)	Overall cost per prisoner (£)]
HMP A	15.2.15-7.7.15	175	24	2625	172.87	26	1055	40.5	£35.17	6478	249.17
HMP B	15.2.15-7.7.15	175	24	2625	172.87	26	1055	40.5	£35.17	6478	249.17
HMP C	11.12.15-26.2.16	18	2	270	207.6	-	-	-	-	415.20	0
HMP D	23.6.16-9.8.16	8	2	120	132.6	4	90	22.5	£28.87	500.7	125.17

For peer review only - http://bmjopen.bmj.com/site/about/guidelines.xhtml

Appendix K: Figure three prisoner ACCTs, intervention and post assessment relative to baseline assessment*

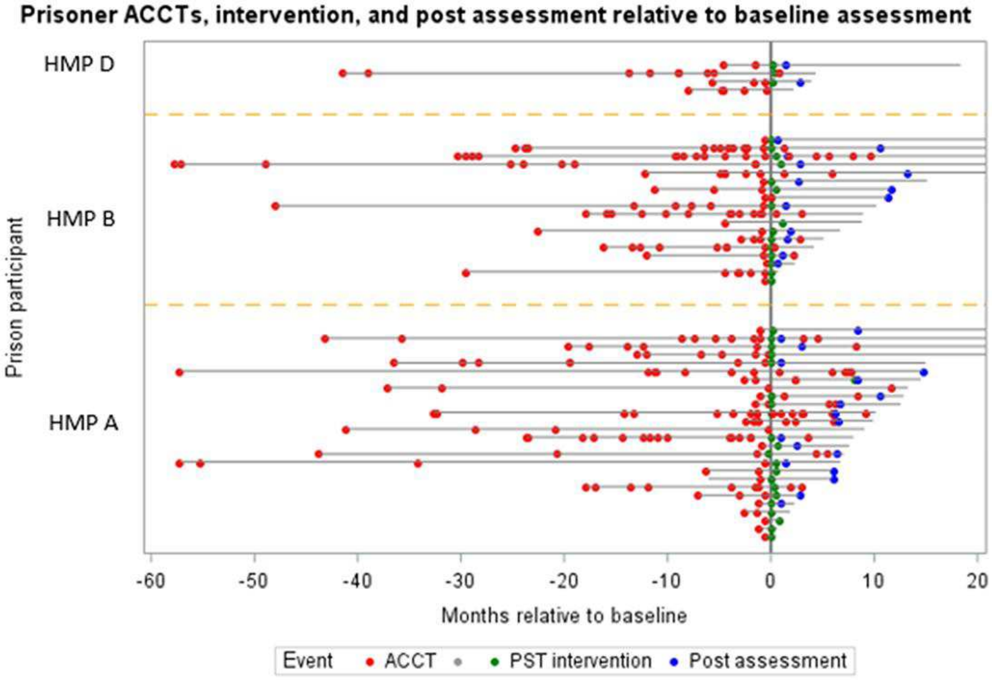

*Note that ACCT data were available up to 5 months in HMP D, 12 months in HMP B, and 13 months in HMP A.

Appendix L: Figure four Phq-9 score at baseline and follow-up

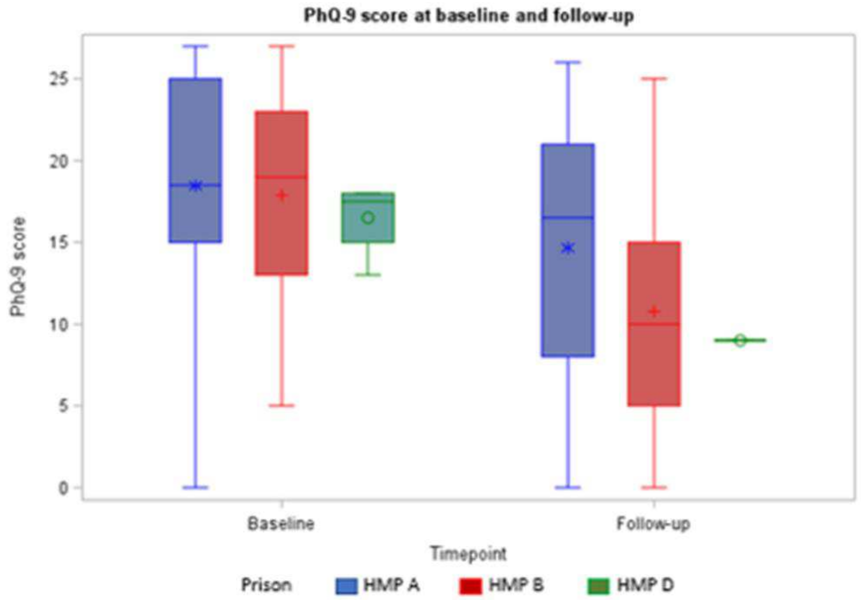

Note: follow-up took place a median of 2.8 months post-baseline, between 3 weeks and 15 months.

13

BMJ Open

Problem-Solving Training: assessing the feasibility and acceptability of delivering and evaluating a problem-solving training model for front-line prison staff and prisoners who self-harm.

Journal:	*BMJ Open*
Manuscript ID	bmjopen-2018-026095.R1
Article Type:	Research
Date Submitted by the Author:	24-Apr-2019
Complete List of Authors:	perry, Amanda; Unviersity of York, Health Sciences; University of York<br>Waterman, Mitchell; University of Leeds, Faculty of Medicine and Health<br>House, Allan; Academic Unit of Psychiatry,<br>Wright-Hughes, Alexandra; University of Leeds, Clinical Trials Research Unit<br>Greenhalgh, Joanne; University of Leeds, Sociology and Social Policy<br>Farrin, Amanda; University of Leeds, Clinical Trials Research Unit<br>Richardson, Gerry; University of York, Centre for Health Economics<br>Hopton, Ann; Universiy of York, Health Sciences<br>Wright, Nat; Spectrum Community Health CIC
**Primary Subject Heading**:	Mental health
Secondary Subject Heading:	Health economics, Public health, Medical education and training
Keywords:	Prisoners, Suicide & self-harm < PSYCHIATRY, Feasibility, EDUCATION & TRAINING (see Medical Education & Training)

SCHOLARONE™
Manuscripts

Title: Problem-Solving Training: assessing the feasibility and acceptability of delivering and evaluating a problem-solving training model for front-line prison staff and prisoners who self-harm.

AUTHORS:

Amanda E. Perry[1]

Mitch G. Waterman[2]

Allan O. House[3]

Alexandra Wright-Hughes[4]

Joanne Greenhalgh[5]

Amanda Farrin[4]

Gerry Richardson[6]

Ann Hopton[1]

Nat Wright [7]

Corresponding authors email address: amanda.perry@york.ac.uk

[1] Department of Health Sciences, University of York
[2] Faculty of Medicine and Health, University of Leeds, Leeds LS2 9JT, UK
[3]Leeds Institute of Health Sciences, University of Leeds, Leeds LS2 9JT, UK
[4] Clinical Trials Research Unit, Leeds Institute of Clinical Trials Research, University of Leeds, Leeds LS2 9JT, UK
[5] Sociology and Social Policy, Faculty of Education and Social Science and Law, University of Leeds, LS2 9JT, UK.
[6] Centre for Health Economics, University of York, York, YO10 5DD Sociology and Social Policy, Faculty of Education and Social Science and Law, University of Leeds, LS2 9JT, UK.
7. Spectrum Community Health CIC, Wakefield, WF1 5RH.

For peer review only
For peer review only - http://bmjopen.bmj.com/site/about/guidelines.xhtml

ABSTRACT

Objectives: Problem-solving skills training is adaptable, inexpensive and simple to deliver. However, its application with prisoners who self-harm is unknown. The study assessed the feasibility and acceptability of a Problem-Solving Training (PST) intervention for prison staff and prisoners who self-harm, to inform the design of a large-scale study.

Design and setting: A mixed methods design used routinely collected data, individual outcome measures, an economic protocol and qualitative interviews at four prisons in Yorkshire and Humber, UK.

Participants: (i) front-line prison staff, (ii) male and female prisoners with an episode of self-harm in the previous two weeks.

Intervention: The intervention comprised a one-hour staff training session and a 30-minute prisoner session using adapted workbooks and case studies.

Outcomes: We assessed the study processes - coverage of training; recruitment and retention rates and adequacy of intervention delivery - and available data (completeness of outcome data, integrity of routinely collected data and access to NHS resource information). Prisoner outcomes assessed incidence of self-harm, quality of life and depression at baseline and at follow-up. Qualitative findings are presented elsewhere.

Results: Recruitment was higher than anticipated for staff n=280, but lower for prisoners, n=48. Retention was good with 43/48 (89%) prisoners completing the intervention, at follow-up we collected individual outcome data for 34/48 (71%) of prisoners. Access to routinely collected data was inconsistent. Prisoners were frequent users of NHS healthcare. The additional cost of training and intervention delivery was deemed minimal in comparison to ‘treatment as usual’. Outcome measures of self-harm, quality of life and depression were found to be acceptable.

Conclusions: The intervention proved feasible to adapt. Staff training was delivered but on the whole it was not deemed feasible for staff to deliver the intervention. A large-scale study is warranted, but modifications to the implementation of the intervention are required.

STRENGTHS AND LIMITATIONS OF THIS STUDY

Prison staff and prisoners were involved in the development of our questionnaires, the intervention adaptation and production of the workbooks.

The feasibility study was conducted across four prison sites including male and female prisoners.

Outcome data were collected via a variety of different sources demonstrating variability and differences in data collection procedures.

It was not deemed feasible for staff to deliver the intervention.

For peer review only - http://bmjopen.bmj.com/site/about/guidelines.xhtml

INTRODUCTION

Problem-solving skills training delivered in a systematic manner provide a non-specialist intervention that is accessible to anyone following brief training. Deficits in problem-solving skills are often found in people who self-harm and can result in reliance on others, leading to a passive as opposed to an active problem-solving approach [1-3]. Problem solving skills have been used in a variety of different contexts and most recently are promoted by The World Health Organisation as 'Problem Management Plus' (PM+ [4]) . They refer to their scheme as a simplified, scalable intervention, in that their delivery requires a less intensive level of specialist human resource use [5]. Trials of problem-solving skills in the *community* demonstrate that teaching people to use brief problem-solving skills can reduce repetition of self-harm behaviour [6-8].

In prison, despite growing numbers of those who self-harm there is a lack of psychological support for prisoners and a recognised need to provide adequate staff training (NICE Guidance CG133: https://www.nice.org.uk/guidance/cg133/chapter/2-Research-recommendations). Evaluations of trials in prisons have explored alternative therapy models for those who self-harm (e.g., cognitive behaviour therapy and interpersonal psychotherapy), but such interventions require the use of extensive resources, large numbers of therapy sessions and qualified clinical therapists, making them inaccessible for prisoners who might only be incarcerated for short periods of time [9, 10].

Use of a brief PST intervention offers one solution to this problem. It has the advantages of being deliverable by any member of staff making it an attractive, inexpensive opportunity to reduce repeat self-harm. However, it is unclear whether the training is acceptable, or whether it can be implemented by staff in this setting. We therefore assessed the feasibility and acceptability of adapting an existing PST for frontline prison staff with the intention that they would deliver the intervention to prisoners who self-harm. This article reports on the acceptability and feasibility of the training, and the implementation of the intervention. Detailed methods on the qualitative findings are elsewhere [11].

MATERIALS AND METHODS

Study design and setting

The study used a mixed-methods design - including quantitative collection of routine data, individual outcome measures and economic resource data, and information from staff to identify how much time was spent on 'usual care'.

The study took place in four prisons in Yorkshire and Humber between September 2014 and May 2017. The study sites included two male adult local prisons where the majority of prisoners are awaiting sentence (housing up to 1,212 and 1052 prisoners, prisons A and B), one female prison (housing up to 416: prison C) and one resettlement prison where sentenced prisoners are housed prior to transfer (housing up to 825: prison D).We report on our intervention using the template for intervention description and replication (TIDier) checklist [12].

Patient and Public Involvement

Our research questions and outcome measures were not informed by prisoner preferences and prisoners were not involved in the recruitment to the study. Prisoners did contribute significantly to the format and adaptation of the training materials. The training materials

For peer review only - http://bmjopen.bmj.com/site/about/guidelines.xhtml

were printed from within the prison by prisoners. The results were disseminated using an A4 summary sheet which was sent to prisoners and prison staff.

The intervention

The PST intervention that we adapted for use in our study was originally devised in New Zealand for patients who self-harm in the community [13]. The theory behind social problem-solving is well established and often forms part of more extensive cognitive behaviour therapy sessions [14, 15]. The seven-step model includes 'getting the right attitude' (step one), reflection and recognising triggers (step two), defining a clear problem (step three), brain storming solutions (step four), decision making (step five), making a plan (step six) and reviewing progress (step seven). Problem-solving skills are an approach that encourages an individual to address their problems in a proactive manner using the systematic seven-step process.

The adaptation of the training and intervention materials

The training was adapted using focus groups. They were used to ensure (i) the appropriateness and context of the case materials and (ii) to promote discussion with staff and prisoners about their views on how the study might work. The refinement process involved a series of structured discussions facilitated by the research team to inform literacy levels in the population and scenario situations that could be used in training as examples of people that staff and prisoners could recognise and/or deal with on a regular basis.

Staff training and recruitment

Staff were recruited with the help of prison liaison staff who assisted with room bookings, shift management and allocation of individuals to attend the training course. Using estimates provided by the prison about: the number, and type of staff employed by the prison, we estimated a feasible recruitment goal of 125 trained staff across the four sites in our 12-month training period.

Staff received a one-hour training session between March 2015 and August 2016. Training was delivered by the research team in a flexible manner (e.g., during induction or on a lunchtime). Eligible prison staff included anyone with responsibility for prisoners who were at risk of self-harm and who were monitored under the prison system (Assessment Care in Custody Teamwork: ACCT[16]). Invited staff groups included management, probation, teaching, prison officers, chaplaincy, psychologists, specialist suicide prevention assessors and nursing staff. All staff receiving the training gave full informed consent.

Recruitment and implementation of the intervention with prisoners who self-harmed

Recruitment of prisoners occurred at prison sites A, B and D. In site C access to the prison site was limited. Our feasible recruitment goal of 120 were based on access to three sites and monthly prison information on the numbers of those 'at risk'.

Prisoners were identified via the ACCT register and approached by a member of the research team or prison staff. Eligible prisoners were 1) >16 years or over and (2) with an episode of self-harm or attempted suicide in the previous two weeks. Prisoners were excluded if (i) an ACCT was opened for reasons other than actual self-harm or attempted suicidal behaviour, (ii) they were deemed too unwell by prison staff, or (iii) posed a risk to the researchers. Consenting participants completed baseline and follow-up questionnaires.

For peer review only - http://bmjopen.bmj.com/site/about/guidelines.xhtml

The entirety of the intervention was delivered in a 30-minute session. The session demonstrated use of the seven-steps using the booklets and case studies developed in the focus groups. Prisoners were invited to attend subsequent follow-up sessions to assess progress and support their engagement with the intervention.

Feasibility and acceptability measures

Data were collected on rates of recruitment, consent and retention for staff and prisoners. Reasons for non-participation and withdrawal were collected, where possible.

For outcome measures we assessed feasibility and acceptability by recording completion and follow-up rates. Typically, completion rates <50% are taken to indicate non-feasibility, >75% as indicating feasibility, and 51-74% as ambiguous - requiring modifications to design or implementation plans and reconsideration.

Our primary outcome proposed for a definitive trial was incidence of self-harm. Data on self-harm and/or attempted suicides were recorded at three months prior to baseline, baseline, post-intervention and at three months follow-up (or up to point of release or transfer) from SystmOne using the search terms 'self-harm' and 'F213'[7]. We explored recording of self-harm incidents through the prison ACCT register but found inconsistencies in the coding of data across the four sites.

Individual secondary outcomes at baseline and follow-up included measurement of quality of life using the EQ-VAS: [17] and depression using the PHQ-9:[18]. The EQ-VAS is a self-rated questionnaire providing description of the subject's current health in five dimensions i.e., mobility, self-care, usual activities, pain/discomfort and anxiety/depression and is rated into one of three degrees of disability (severe, moderate or none). The PHQ-9 is a well validated tool for the measurement of depression with robust psychometric properties, reliability and validity in adult community populations.

Cost of usual care were estimated by: (i) completion of a self-report questionnaire reporting on access to NHS treatment before, during and after the study, (ii) staff interviews to ascertain the average time spent on each ACCT process and (iii) a case note review of eleven prisoner ACCT documents to record the amount of staff time involved in the ACCT procedure.

The costs of training included (i) the costs to release staff in attending the training sessions, (ii) the facilitator time in the delivery of the training and (iii) the number of training sessions, numbers of staff attending each session, and the duration and timing of each training session.We obtained routinely collected electronic ACCT data consisting of individual and monthly ACCT information between January 2012 and December 2016. The time period of the data collection was prescribed by the individual prison data collection protocols (Appendix A supplementary materials). We found that data were comparable from our four prison sites across this time period. Prior to 2012 the comparability of data and access to data were found to be limited and December 2016 was the latest date for which all prisons had complete data.

DATA ANALYSIS

[7] F213 is the title of the form used by the prison service to record incidents of self-harm behaviour

For peer review only - http://bmjopen.bmj.com/site/about/guidelines.xhtml

Data were summarised, by prison, using descriptive summary statistics. The information included the description of the focus group participants, the number of training sessions and staff attending training sessions. The feasibility and success of recruitment of prisoners to the study is evaluated through summaries of the screening, eligibility, consent and recruitment processes.

A summary of the variability of available routine data across: outcomes, prison and wings (where available), and the estimated cost of usual care were informed using staff information and case review process. Delivery and implementation of the PST intervention were estimated using the numbers of training sessions, numbers of staff attending, standardised staff costs, facilitator time in the delivery of the session and preparation for each session alongside the cost of materials. Summary statistics for prisoner's baseline characteristics and outcomes for the incidence of self-harm behaviour, quality of life, depression and information on access to NHS treatment were recorded.

RESULTS

Feasibility assessment

Adapting and developing the materials

During 2015 staff and prisoners were nominated by each prison to participate in focus groups. 31 staff participants attended (Appendix B supplementary materials). They comprised of mainly operational 17 /31(55%) or managerial 6/31 (19%) staff with a mean age of 37 years. The majority were female 20/31 (66%), spoke English as their first language 27/31 (88%) and were British 27/31 (90%).

Six focus groups involving 67 prisoners, included mainly male prisoners 56/67 (83.6%) with a mean age of 39.8 years (SD 9.63). There were fewer prisoners on remand or first-time offenders involved in the focus groups, compared to recruited prisoners for the study (Appendix C supplementary materials). The process resulted in two gender-specific picture booklets and a series of exercises with associated case study scenarios that were used in the training and delivery of the intervention.

Coverage of staff training and recruitment

280 prison staff were trained between March 2015 and August 2016 (see figure 1). Training was delivered by the research team to staff groups with a mean size of 8 staff (range of group sizes 2-19). Recruitment of staff to training sessions appeared to be acceptable and feasible.

[Insert Figure 1 here]

Staff trained were mainly operational (120, 43%) or healthcare staff (78, 28%); other staff included a number of voluntary, managerial, admin, education, and offender manager probation staff. Mean age of staff trained was 42 years, 59% were male, and almost all spoke English as their first language and were British. Trained staff had spent a median of 8 years (range <1 month – 36 years) working in the prison service (see table 1).

[Insert table 1 here]

Screening and recruitment of prisoners

During the three-month recruitment period at each site a total of 281 prisoners were eligible to participate as per the study criteria. Of these, 106/281 (37%) were released or transferred

For peer review only - http://bmjopen.bmj.com/site/about/guidelines.xhtml

to another prison site prior to invitation to attend an appointment in healthcare. The average time between identification of an eligible participant and meeting them to inform them about the studied varied between at each site between one and three weeks.

Of the remaining 175 (62%), 95/175 were not seen in healthcare for a variety of reasons. These included: 66/95 (69%) people who did not attend their appointment to be informed about the study following three consecutive invitations, 9/95 (9%) were considered too dangerous to approach, 6/95 (6%), lacked sufficient capacity, 5/95 (5%), were transferred or released prior to attending the appointment, 8/95 (8%), were not approached by the research team due to limited resources within the team and one person died 1/95 (1%). Of the remaining 83 people, 6 (7%) attending the appointment were deemed not eligible reporting no incident of actual self-harm behaviour. For the remaining 75 people 29/75 (39%) did not consent to take part leaving 48/75 (66%) consenting participants.

[Insert Figure 2 here]

The median age of prisoners was 30 years (range 59 to 58 years). All but three were White British, and all spoke English as their first language. Two thirds 32/48 (67%) were single and had never married; the majority smoked 39/48 (83%) and did not have a physical or learning disability (36/48 77% and 33/48 69%). Only a minority of prisoners recruited from prison B and none of those in prison D were on remand, whilst almost half of prison A recruited prisoners were on remand 22/48 (46%). Only a quarter were first time offenders 12/48 (25%), the number of times prisoners had been in prison ranged up to 50, with a median of 3 times. The median length of sentence was 27 months, with prisoners having spent a median of 3 months (range 2 days to 2 years) in their current and a median of 9 months left in prison (range 3 days to 15 years). For self-harm details see Appendix D supplementary materials.

Retention

5/48 (10%) participants did not complete the intervention and withdrew from the study (figure 2); although general reasons were not provided for withdrawal. We tracked the transfer of 7/48 (15%) prisoners between our study sites. Transfer reasons included the progression of prisoners through their sentence (e.g., from a local prison to our resettlement prison) or were unexpected due to a security breech.

Adequacy of intervention delivery with prisoners who self-harmed

Between August 2015 and June 2016 delivery of the intervention by staff occurred for only two prisoners. At prison C the research team had limited access to deliver the intervention and instead the prison decided to take the booklets and distribute them on the wings to target bullying. For the remaining 46/48 (96%) participants the intervention was delivered by members of the research team in the healthcare unit.

The median time spent on intervention delivery was 40 minutes per prisoner, (range 30-90 minutes). The overall time spent with the researcher, including the baseline assessment, intervention delivery, follow up questionnaire for outcomes and qualitative interview averaged a median of 80 minutes, (range 30 minutes up to 2 hours 30 minutes) over a period of 1-7 contact appointments. In interviews, the intervention was acceptable to prisoners who received the intervention [7].

Acceptability of outcome measures

Use of routinely collected data to inform large-scale study

For peer review only - http://bmjopen.bmj.com/site/about/guidelines.xhtml

For peer review only

We found that reporting of self-harm data was complicated and recorded by several different methods, with variability in recording and differing definitions of self-harm across the four sites (Appendix E supplementary materials). Figure 3 shows the variability in monthly number of ACCTs opened at each site per 100 prisoners. The greatest variability of open ACCTs was displayed in prison C (our female site): Appendices F supplementary materials provide further details.

[Insert Figure 3 here]

Estimating the costs of usual care

Access to NHS services

All 48 prisoners had received some NHS service provision whilst in prison. Access to a GP (range 1-10 appointments), pharmacist (daily drug dispensing) or duty nurse (range 1-35 appointments) appointments were the most cited points of contact. 35/48 (73%) prisoners reported accessing mental health services, two reported access to a psychological therapy. Just under a quarter 11/48 (23%) had experienced a hospital admission and 13/48 (27%) (range 1-9) reported attending accident and emergency in the three months prior to incarceration. In all cases, admissions were related to synthetic cannabinoids intake, overdose, attempted hanging or feeling suicidal. There were no adverse events reported as part of the PST intervention during the study period.

We collected information from staff about how much time they spent conducting each element of the ACCT process (Appendix G supplementary materials). Using an average time spent, each task in the ACCT process was assigned a proportionate salary costs (Appendix H supplementary materials).

We combined this staff information with data that we collected from the case review of eleven prisoners who had been on an ACCT during the study period. The eleven prisoners represented a total of twenty-four ACCTS documents that had been 'open' and 'shut' during their stay within the prison. For two prisoners the ACCT was in use at the point of data collection providing a conservative estimate of cost. We added up the numbers of case reviews for each prisoner which ranged from one to thirty-three, and added up the number of staff observations per ACCT document which ranged between 0 and5520 staff observations. The total administrative costs for the eleven prisoners was estimated at £21,650, an average of £1,968 per prisoner (range £375-£6416).

Estimating the costs of training

Training costs included a notional hourly rate (of £15 per person) to cover the cost of releasing staff to attend the training session, and included the travel, preparation time and facilitator time in delivering the course and the cost of course materials. Across sites we estimated the training costs of between £500 and £6406 equating to a cost per prisoner of between £125 and £246 (Appendix H supplementary material).

Overall it proved feasible to gather resource information to provide a cost estimate of usual care, delivery of training and implementation of the intervention

Prisoner outcomes

For peer review only - http://bmjopen.bmj.com/site/about/guidelines.xhtml

100% of those agreeing to participate in the study completed the baseline assessment. Follow-up times varied considerably, taking place a median of 2.8 months after recruitment but up to a maximum of 15 months for one prisoner (Appendix I supplementary materials). The timing of follow up assessments fell into three clusters, the largest cluster taking place within the first three months post recruitment, a further set taking place between 6 and 8 months post recruitment in prison A. Follow-up was affected when access to prison A was halted for a three-month period (figure 4). Overall the average follow-up rate for questionnaire returns was 34/48 (71%) across the three sites. The changes in scores reflect them as potentially useful outcome measures that could be used in a large-scale evaluation.

[Insert Figure 4 here].

Primary outcome: incidence of self-harm behaviour

Incidence of self-harm behaviour appeared to decrease over the life time of the project. At 3 months prior to baseline, 32/48 (66%) prisoners had harmed themselves. This reduced to 9/48 (18%) prisoners at post-test.

Secondary outcomes

Quality of life

A total of 32/48 (66%) of individuals completed full information on the EQ-VAS. The baseline mean score (0.504, SD 0.34) fell post intervention (0.625, SD, 0.347).

Depression

At baseline, median scores were high at 18 and most prisoners had either moderately severe 18/48 (38%) or severe depression 20/48 (40%). Prisoners' at follow-up had lower depression scores with just 7/48 (15%) classed as moderately severely depressed, and 13/48 (27%) as severely depressed (Appendix J supplementary materials).

DISCUSSION

The study aimed to assess the feasibility and acceptability of adapting and implementing a brief PST intervention for prison staff and prisoners at risk of self-harm. Our results indicate that staff can be trained in using these skills though most were unable to implement them with those who self-harmed. Prison staff faced severe time pressures, and limited resources making it difficult to accommodate the translation of knowledge into practice. This is a common problem in the design and implementation of complex interventions in organisations other than healthcare [19]. These findings emerged during the implementation phase. The brief nature of the training sessions themselves did not, perhaps, facilitate the expression of these doubts or tackle approaches to translation of skills into practice in a pressured environment.

Attrition from the study sample by prisoners was minimal due to the 30-minute intervention design. Previous prison trials have demonstrated relatively high levels of attrition. In our study (despite a lack of access to one site for three months) we managed to produce encouraging follow-up rates (71%) suggesting that our outcomes were acceptable. Our findings are comparable with other pilot trials of self-harm in prisons [9, 10] and trials of

For peer review only - http://bmjopen.bmj.com/site/about/guidelines.xhtml

suicide prevention more broadly in the community [20]. We were able to track participants through our sites. This allowed us to collect follow-up data on seven participants who were released from prison A, and either returned back to the same prison during the study period or were transferred to prison C prior to release. Prison function is therefore an important consideration. Turnover of prisoners at our local prison sites (e.g., prisons A and B) was considerably greater than in our resettlement prison. This finding is supported elsewhere with data provided from prison A in a recent Inspectorate report showing that 430/1109 (38%) were imprisoned for less than three months in 2017. Prisoners followed from prison A through to prison C were notably in a better position to engage with training when in the resettlement prison. This system of 'tracking' participants provides a potential mechanism to ensure adequate follow-up in a large-scale study.

There were limitations with the development of our economic protocol in the assumptions made with regards to costs for usual care which are not necessarily representative. Access, quality and consistency of these data varied across the prison sites and led us to conclude that such routine data could only be used to measure the impact of any future evaluation if additional data were provided or stricter collection protocols and monitoring were deployed. We also propose that any new study should include individual self-report information and information from local and national data sources. This method is not dissimilar to other data collection mechanisms in two pilot trials of self-harm in UK prisons where prisoners report suicidal behaviours, thoughts and feelings [9, 21].

In designing a large-scale study, we have sufficient information to inform our outcomes of measurement and feasibility of data collection. However, alternative implementation mechanisms need to be identified prior to any large-scale study. Our qualitative findings [11] suggest two alternative options: first, use of trusted prisoners as 'problem support mentors' to deliver the skills to peers on the wings and/or second, delivery of problem-solving skills to prisoners through education classes.

CONCLUSIONS

The study suggests that the modified version of PST, adapted for training, was acceptable to prisoners. Although the study demonstrated that it was currently not feasible to deliver the intervention using prison staff it provides insight into how such an intervention with prisoner-staff involvement can be adapted for use in a different environment.

Author Affiliations:

Department of Health Sciences, University of York
Faculty of Medicine and Health, University of Leeds, Leeds LS2 9JT, UK
Leeds Institute of Health Sciences, University of Leeds, Leeds LS2 9JT, UK
Clinical Trials Research Unit, Leeds Institute of Clinical Trials Research, University of Leeds, Leeds LS2 9JT, UK
Centre for Health Economics, University of York, York, YO10 5DD
Spectrum Community Health CIC, Wakefield, WF1 5RH.

Acknowledgements:

The authors are grateful to Spectrum Community Health CIC and previously Leeds Community HealthCare Trust who agreed to host this study. To Christine Butt who helped

For peer review only - http://bmjopen.bmj.com/site/about/guidelines.xhtml

with the collection of data at one prison site. We would also like to acknowledge the contributions of all the prison staff, prisoners, for support from the North East and Yorkshire Area Safer Custody Team and prison Governors for their willingness to take part and support this study.

Contributors:

AP, AH AND MW designed and conducted most of the study with considerable input from AKH. AWH and AF took the lead in performing the statistical analyses together and JG was the lead for analysing the qualitative interviews with AP. GR and NW led the development of the economic protocol and information on the study costs. NW supported access to the prison sites and all authors provided input into the writing of the manuscript.

Funding: This paper presents independent research funded by the NIHR under its Research for Patient Benefit (RfPB) Programme (Grant Reference Number PB-PG-0211-24122).

Disclaimer: The views expressed are those of the author(s) and not necessarily those of the NHS, the NIHR or the Department of Health

Patient consent: Obtained

Competing interests None declared

Ethics approval: Ethical approval for the study was obtained for phase one from NHS REC approval [NRES, North East York, 28.10.14] and NOMS [1.9.14] and phases two-five [Bristol REC Centre, London South East, 6.1.15] from NHS REC approval, NOMS [6.3.15] and the Department of Health Sciences at the University of York for all phases [11.12.14]. As the material was adapted and developed for appropriate use within each prison we were granted one substantive amendment to the project from all parties during July 2015.

Provenance and peer review: Not commissioned; externally peer reviewed.

Data sharing statement: The data is available by contacting the lead author amanda.perry@york.ac.uk. Any data will not contain any unique personal identifiable information about the persons in the database

Figure Legends:

Figure 1: Staff trained and participating focus groups

Figure 2: Flow of study participants through study

Figure 3: Monthly numbers of ACCTs opened per 100 prisoners

Figure 4: Time between recruitment and questionnaire follow-up assessment

References

1. Linehan, M.M., et al., *Interpersonal Problem-Solving and Parasuicide.* Cognitive Therapy and Research, 1987. **11**(1): p. 1-12.
2. McLeavey, B.C., et al., *Interpersonal problem-solving skills training in the treatment of self-poisoning patients.* Suicide Life Threat Behav, 1994. **24**(4): p. 382-94.

For peer review only

3. Pollock, L. and J. Williams, *Effective problem solving in suicide attempters depends on specific autobiographical recall.* Suicide and Life Threatening Behaviour,, 2011. **31**: p. 386-396.
4. Organization., W.H., *Problem Management Plus (PM+): Individual psychological help for adults impaired by distress in communities exposed to adversity. (Generic field-trial version 1.1). Geneva, WHO, 2016.*

. 2016.
5. Sijbrandij, M., Farooq, S., Bryant, R. A., Dawson, K., Hamdani, S. U., Chiumento, A., Minhas, F., Saeed, K., Rahman, A., van Ommeren, M. , *Problem Management Plus (PM+) for common mental disorders in a humanitarian setting in Pakistan; study protocol for a randomised controlled trial (RCT).* . BMC Psychiatry, 2015. **15**: p. 232 doi:10.1186/s12888-015-0602-y.
6. Hawton, K., et al., *Evaluation of out-patient counselling compared with general practitioner care following overdoses.* Psychol Med, 1987. **17**(3): p. 751-61.
7. Perry, A., M. Waterman, and A. House, *Problem-Solving Training for Suicidal Prisoners*, in *The Prevention of Suicide in Prison: Cognitive Behavioural Approaches*, P. D, Editor. 2015, Taylor & Francis Ltd: UK. p. 69-83.
8. Hawton, K., et al., *Psychosocial interventions for self-harming adults.* 2016, DOI: 10.1002/14651858.CD012189.: Cochrane Database of Systematic Reviews 2016, Issue 5. Art. No.: CD012189.
9. Walker, T., et al., *The WORSHIP II study: a pilot of psychodynamic interpersonal therapy with women offenders who self-harm.* The Journal of Forensic Psychiatry & Psychology, 2017: p. 1-14.
10. Pratt, D., et al., *Cognitive Behavioural Suicide Prevention for Male Prisoners: A*

pilot randomised controlled trial. Psychological Medicine, 2015. **45**(16): p. 3441-3451.
11. Perry A E., Waterman, M.G., House, A.O., Greenhalgh, J. (2019), *A qualitative examination of problem solving skills in prison: the implementation of training and intervention delivery with frontline prison staff and prisoners who self-harm.* Health and Justice, Special Edition in Suicide and Self-Harm, 2019 in press.
12. Hoffmann, T.C., et al., *Better reporting of interventions: template for intervention description and replication (TIDier) checklist and guide.* British Medical Journal, 2014. **348:g 1687**(doi:10.1136/bmj.g1687.): p. 1-12.
13. Collinson, M., et al., *MIDSHIPS: Multicentre Intervention Designed for Self-Harm using Interpersonal Problem-Solving:protocol for a randomised controlled feasibility study.* Trials, 2014. **15**(163): p. 1-7.

14. Evans, K., et al., *Manual Assisted Cognitive Behavioural Therapy in the Treatment of Recurrent Deliberate Self Harm: A Randomised Controlled Trial.* . Psychological Medicine, 1999. **29**: p. 19-25.
15. D'Zurilla, T.J. and M.R. Goldfried, *Problem solving and behavior modification.* J Abnorm Psychol, 1971. **78**(1): p. 107-26.
16. UK Ministry of Justice, *Management of prisoners at risk of harm to self, to others and from others (safer custody)*, P.S.I. 2011-64., Editor. 2013, http://www.justice.gov.uk/offenders/psis/prison-serviceinstructions- 2011 (accessed Nov 11, 2013).
17. Gusi, N., P.R. Olivares, and R. Rajendram, *The EQ-5D Health-Related Quality of Life Questionnaire*, in *Handbook of Disease Burdens and Quality of Life Measures*, V.R. Preedy and R.R. Watson, Editors. 2010, Springer New York: New York, NY. p. 87-99.
18. Kroenke, K., R.L. Spitzer, and J.B.W. Williams, *The PHQ-9: Validity of a Brief Depression Severity Measure.* Journal of General Internal Medicine, 2001. **16**(9): p. 606-613.
19. Batalden, M., et al., *Co production of healthcare services.* BMJ Qual Saf, 2015. **0**: p. 1-9.
20. Tarrier, N., K. Taylor, and P. Gooding, *Cognitive-behavioral interventions to reduce suicide behavior: a systematic review and meta-analysis.* Behav Modif, 2008. **32**(1): p. 77-108.

For peer review only - http://bmjopen.bmj.com/site/about/guidelines.xhtml

21. Hawton K, O'Connor RC, and S. KEA., *Suicidal behaviour and self-harm. In Thapar A, Pine DS, Leckman JF, et al, eds. Rutter's Child and Adolescent Psychiatry 6th edn. Chichester: Wiley-Blackwell,* . Vol. 893-910.

. 2015.

For peer review only

Table 1: Demographic information of staff trained

	PrisonA (n=175)	PrisonB (n=79)	PrisonC (n=18)	PrisonD (n=8)	Total (n=280)
**Time working in the prison service (Years)**					
N	172	78	18	7	275
Mean (SD)	8.5 (8.93)	13.0 (9.04)	12.9 (8.45)	12.1 (9.91)	10.1 (9.16)
Median (Range)	6.0 (0.0, 36.0)	11.8 (0.1, 35.3)	12.5 (0.5, 25.0)	10.5 (1.3, 29.2)	8.0 (0.0, 36.0)
**Time working in this prison (Years)**					
N	172	78	18	7	275
Mean (SD)	6.2 (7.48)	11.2 (8.15)	7.9 (7.61)	9.0 (7.24)	7.8 (7.96)
Median (Range)	3.3 (0.0, 31.0)	10.9 (0.1, 35.3)	4.5 (0.3, 24.3)	7.9 (1.1, 20.8)	5.5 (0.0, 35.3)
**Since working here have you encountered an incident of self-harm?**					
Yes	119 (68.0%)	68 (86.1%)	18 (100.0%)	8 (100.0%)	213 (76.1%)
No	52 (29.7%)	11 (13.9%)	0 (0.0%)	0 (0.0%)	63 (22.5%)
Missing	4 (2.3%)	0 (0.0%)	0 (0.0%)	0 (0.0%)	4 (1.4%)
**Most recent self-harm incident?**					
Within the past 7 days	44 (37.0%)	28 (41.2%)	7 (38.9%)	3 (37.5%)	82 (38.5%)
Within the past month	24 (20.2%)	17 (25.0%)	4 (22.2%)	0 (0.0%)	45 (21.1%)
Two months or more	20 (16.8%)	8 (11.8%)	2 (11.1%)	1 (12.5%)	31 (14.6%)
Missing	31 (26.1%)	15 (22.1%)	5 (27.8%)	4 (50.0%)	55 (25.8%)
**Type of incident?**					
Self-poisoning	10 (8.4%)	1 (1.5%)	0 (0.0%)	1 (12.5%)	12 (5.6%)
Self-injury	94 (79.0%)	61 (89.7%)	16 (88.9%)	6 (75.0%)	177 (83.1%)
Mixed self-poisoning and self-injury	7 (5.9%)	4 (5.9%)	2 (11.1%)	1 (12.5%)	14 (6.6%)
Suicide	7 (5.9%)	2 (2.9%)	0 (0.0%)	0 (0.0%)	9 (4.2%)
Missing	1 (0.8%)	0 (0.0%)	0 (0.0%)	0 (0.0%)	1 (0.5%)
**Attended self-harm training?**					
Yes	74 (42.3%)	48 (60.8%)	13 (72.2%)	3 (37.5%)	138 (49.3%)
No	96 (54.9%)	30 (38.0%)	3 (16.7%)	5 (62.5%)	134 (47.9%)
Cannot recall	4 (2.3%)	1 (1.3%)	2 (11.1%)	0 (0.0%)	7 (2.5%)
Missing	1 (0.6%)	0 (0.0%)	0 (0.0%)	0 (0.0%)	1 (0.4%)
**Time since self-harm training?**					
N	66	45	13	3	127
Mean (SD)	30.5 (38.22)	20.4 (27.32)	41.7 (53.84)	19.0 (20.42)	27.8 (36.64)
Median (Range)	12.0 (0.0, 180.0)	12.0 (1.0, 120.0)	12.0 (1.0, 168.0)	12.0 (3.0, 42.0)	12.0 (0.0, 180.0)
**Who provided this training?**					
Prison service	59 (79.7%)	42 (87.5%)	8 (61.5%)	3 (100.0%)	112 (81.2%)
NHS	3 (4.1%)	0 (0.0%)	2 (15.4%)	0 (0.0%)	5 (3.6%)
Nurse Training	2 (2.7%)	1 (2.1%)	0 (0.0%)	0 (0.0%)	3 (2.2%)

For peer review only - http://bmjopen.bmj.com/site/about/guidelines.xhtml

	PrisonA (n=175)	PrisonB (n=79)	PrisonC (n=18)	PrisonD (n=8)	Total (n=280)
Other including University	3 (4.1%)	2 (4.2%)	1 (7.7%)	0 (0.0%)	6 (4.3%)
Missing	7 (9.5%)	3 (6.3%)	2 (15.4%)	0 (0.0%)	12 (8.7%)
**Length of training?**					
1 hour	19 (25.7%)	0 (0.0%)	1 (7.7%)	0 (0.0%)	20 (14.5%)
2 hours	9 (12.2%)	4 (8.3%)	1 (7.7%)	1 (33.3%)	15 (10.9%)
Half day	16 (21.6%)	14 (29.2%)	1 (7.7%)	1 (33.3%)	32 (23.2%)
Full day	13 (17.6%)	18 (37.5%)	7 (53.8%)	1 (33.3%)	39 (28.3%)
More than one day	7 (9.5%)	4 (8.3%)	3 (23.1%)	0 (0.0%)	14 (10.1%)
Missing	10 (13.5%)	8 (16.7%)	0 (0.0%)	0 (0.0%)	18 (13.0%)

For peer review only

Figure 1: Staff trained and participating focus groups

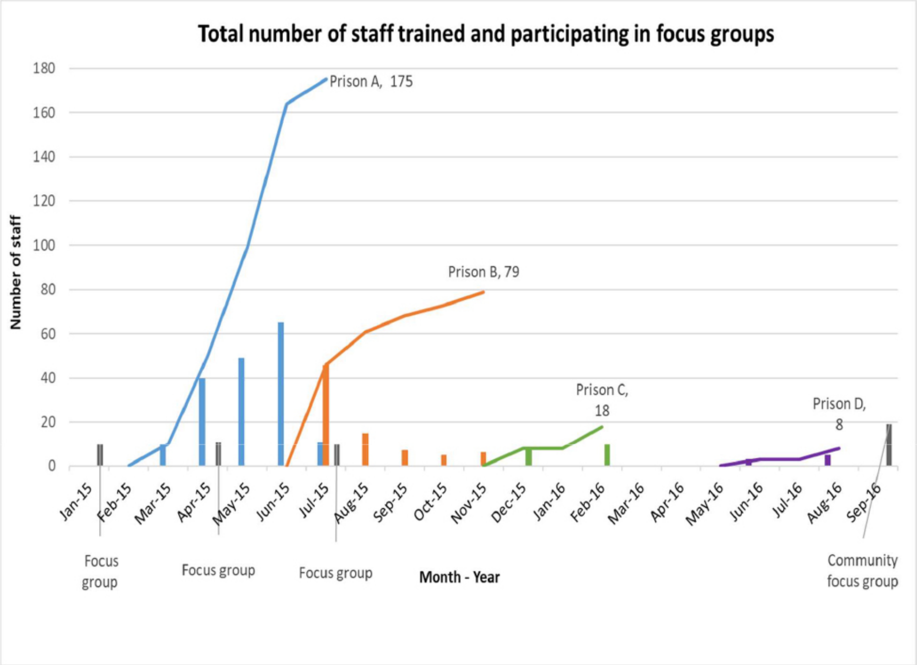

Figure 1: Staff trained and participating focus groups

90x90mm (300 x 300 DPI)

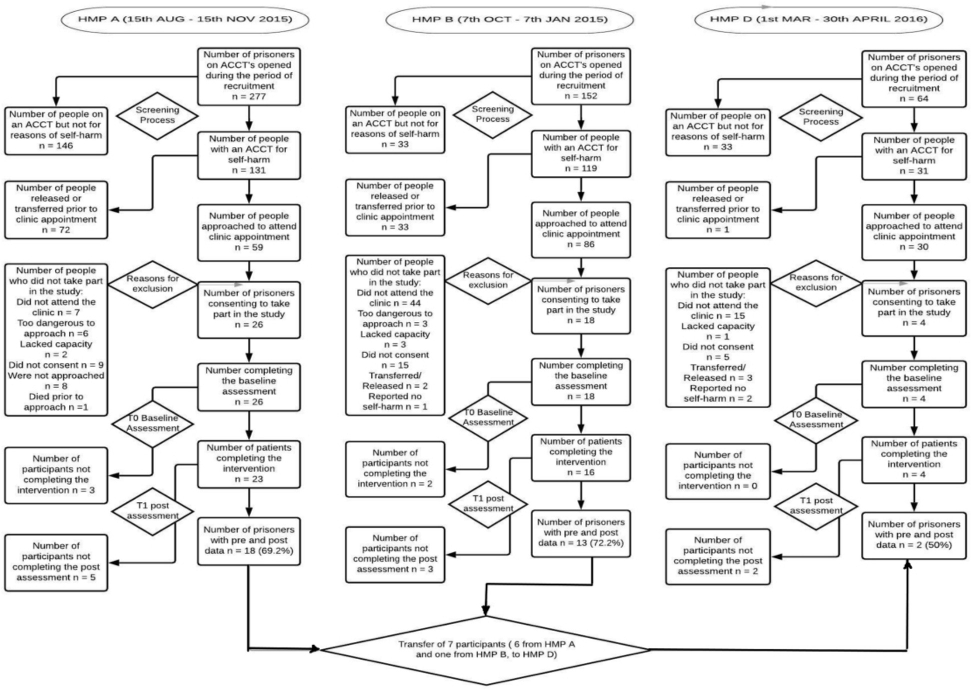

Figure 2

210x150mm (300 x 300 DPI)

Figure 3: Monthly numbers of ACCTs opened per 100 prisoners

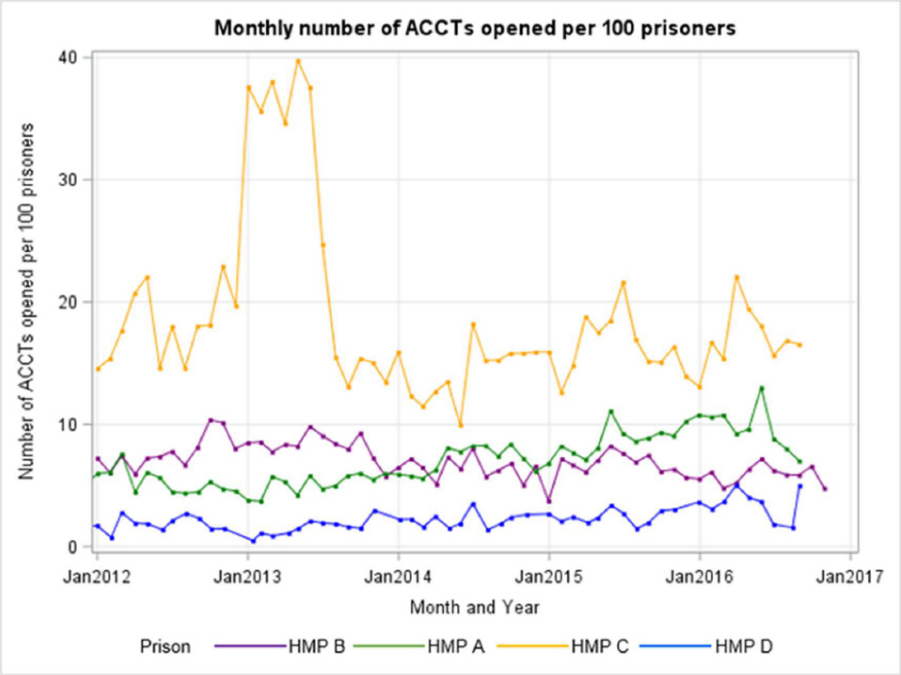

Figure 3: Monthly numbers of ACCTs openned per 100 prisoners

90x90mm (300 x 300 DPI)

Figure 4: Time between recruitment and questionnaire follow-up assessment

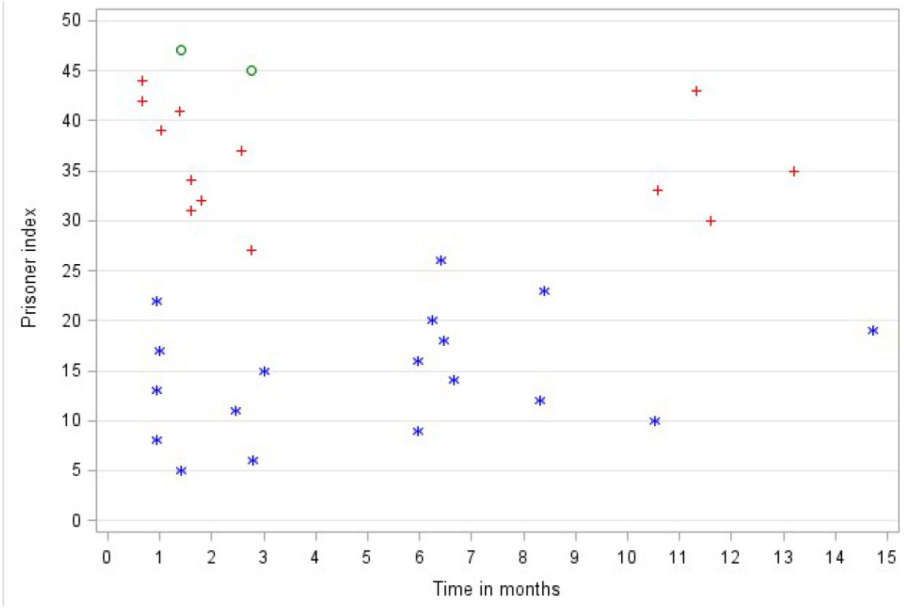

Key: * = HMP A, + = HMP B, o = HMP D.

Figure 4: Time between recruitment and questionnaire follow-up assessment

90x90mm (300 x 300 DPI)

Web Appendix Supplementary Materials

Appendix A: Table one data collection protocols

Appendix B: Table two prison staff focus group participation

Appendix C: Table three prisoner focus group participation

Appendix D: Table four prisoner self-harm details

Appendix E: Table five coding variability across prison sites

Appendix F: Figure 1 numbers of prisoners at unlock on last day of the month

Appendix G: Table six standardised ACCT process costs

Appendix H: Table seven training and implementation costs

Appendix I: Figure 2 prisoner ACCTs, intervention and post assessment relative to baseline assessment

Appendix J: Figure 3 Phq-9 score at baseline and follow-up

For peer review only

Appendix A: Table one data collection protocols

Prison Site	Prison A	Prison B	Prison D	Prison C
**Frequency of ACCTs per year**				
2009			61	
2010			118	
2011	754		168	
2012	756	840	170	779
2013	730	734	154	718
2014	1012	645	208	688
2015	1219	798	249	729
2016 – partial*	1010	675	262	605
**Number of ACCTs per year (2012 - 2015)**				
Mean (SD)	929.3 (231.31)	754.3 (84.88)	195.3 (42.39)	728.5 (37.86)
Median (Range)	884 (730, 1219)	766 (645, 840)	189 (154, 249)	724 (688, 779)

*2016 frequencies up to September in Prison A, D and Cl, and up to November in Prison B.

For peer review only - http://bmjopen.bmj.com/site/about/guidelines.xhtml

Appendix B: Table two prison staff focus group participation

	PrisonA (n=10)	PrisonB (n=11)	PrisonC (n=10)
**Type of staff**			
Operational staff	7 (70.0%)	7 (63.6%)	3 (30.0%)
Managerial staff	0 (0.0%)	1 (9.1%)	5 (50.0%)
Healthcare staff	0 (0.0%)	1 (9.1%)	2 (20.0%)
Visitor to the prison	2 (20.0%)	0 (0.0%)	0 (0.0%)
Admin / Probation	1 (10.0%)	1 (9.1%)	0 (0.0%)
Missing	0 (0.0%)	1 (9.1%)	0 (0.0%)
**Gender**			
Male	4 (40.0%)	5 (45.5%)	3 (30.0%)
Female	6 (60.0%)	6 (54.5%)	7 (70.0%)
**Age**			
N	10	10	9
Mean (SD)	33.7 (10.07)	42.7 (11.41)	46.9 (6.15)
Median (Range)	33.0 (19, 49)	44.0 (21, 58)	47.0 (36, 56)
**First language**			
English	8 (80.0%)	11 (100.0%)	10 (100.0%)
Hungarian	0 (0.0%)	0 (0.0%)	0 (0.0%)
German	0 (0.0%)	0 (0.0%)	0 (0.0%)
Missing	2 (20.0%)	0 (0.0%)	0 (0.0%)
**Ethnic group**			
British	10 (100.0%)	11 (100.0%)	9 (90.0%)
Irish / Other white background	0 (0.0%)	0 (0.0%)	0 (0.0%)
White and Black Caribbean	0 (0.0%)	0 (0.0%)	0 (0.0%)
Indian / Pakistani	0 (0.0%)	0 (0.0%)	1 (10.0%)
**Religious preference**			
No religion	2 (20.0%)	6 (54.5%)	1 (10.0%)
Christian	8 (80.0%)	5 (45.5%)	8 (80.0%)
Muslim	0 (0.0%)	0 (0.0%)	1 (10.0%)
Hindu	0 (0.0%)	0 (0.0%)	0 (0.0%)
**Consider yourself disabled?**			
Yes	0 (0.0%)	0 (0.0%)	0 (0.0%)
No	10 (100.0%)	11 (100.0%)	10 (100.0%)
**Highest academic qualification?**			
Post Graduate	0 (0.0%)	0 (0.0%)	0 (0.0%)
Graduate	5 (50.0%)	5 (45.5%)	2 (20.0%)
A Level or equivalent	5 (50.0%)	3 (27.3%)	7 (70.0%)
GCSE or equivalent	0 (0.0%)	3 (27.3%)	1 (10.0%)

Appendix C: Table three prisoner focus group participation

	PrisonA (n=13)	PrisonB (n=43)	PrisonC (n=11)	Total (n=67)
**Are you on Remand?**				
Yes	2 (15.4%)	2 (4.7%)	1 (9.1%)	5 (7.5%)
No	11 (84.6%)	38 (88.4%)	10 (90.9%)	59 (88.1%)
Missing	0 (0.0%)	3 (7.0%)	0 (0.0%)	3 (4.5%)
**First time offender?**				
Yes	4 (30.8%)	19 (44.2%)	7 (63.6%)	30 (44.8%)
No	9 (69.2%)	23 (53.5%)	4 (36.4%)	36 (53.7%)
Missing	0 (0.0%)	1 (2.3%)	0 (0.0%)	1 (1.5%)
**Age first entered prison (years)**				
N	13	42	11	66
Mean (SD)	21.3 (7.61)	30.0 (13.13)	40.5 (12.71)	30.0 (13.35)
Median (Range)	18.0 (15.0, 40.0)	27.0 (13.0, 61.0)	39.0 (18.0, 60.0)	26.0 (13.0, 61.0)
**Number of times in prison?**				
N	13	40	11	64
Mean (SD)	7.2 (8.67)	2.8 (3.12)	1.2 (0.40)	3.4 (4.94)
Median (Range)	4.0 (0.0, 30.0)	1.5 (1.0, 15.0)	1.0 (1.0, 2.0)	1.0 (0.0, 30.0)
**Time spent in this prison? (months)**				
N	13	42	11	66
Mean (SD)	9.2 (10.66)	29.0 (25.27)	34.8 (26.98)	26.0 (24.71)
Median (Range)	6.0 (1.0, 42.0)	24.0 (2.0, 102.0)	24.0 (11.0, 84.0)	16.0 (1.0, 102.0)
**Length of sentence (months)**				
N	11	38	11	60
Mean (SD)	167.5 (344.02)	208.5 (231.48)	181.9 (108.94)	196.1 (236.56)
Median (Range)	30.0 (18, 1188)	120.0 (8, 666)	168.0 (42, 333)	126.0 (8, 1188)
**Months left until sentence expiry**				
N	9	30	8	47
Mean (SD)	36.1 (44.62)	64.0 (59.95)	76.5 (56.46)	60.8 (57.15)
Median (Range)	16.3 (3.7, 120.2)	41.0 (0.8, 257.9)	56.4 (6.2, 154.6)	40.2 (0.8, 257.9)

For peer review only - http://bmjopen.bmj.com/site/about/guidelines.xhtml

Appendix D: Table four prisoner self-harm details

	PrisonA (n=26)	PrisonB (n=18)	Prison D (n=4)	Total (n=48)
**Ever harmed yourself?**				
Yes	26 (100.0%)	18 (100.0%)	4 (100.0%)	48 (100.0%)
**Time since most recent self-harm (months)**				
N	26	16	4	46
Mean (SD)	1.0 (0.72)	0.7 (0.45)	1.5 (1.86)	1.0 (0.79)
Median (Range)	1.0 (0.0, 3.0)	0.7 (0.0, 1.5)	0.8 (0.1, 4.2)	0.7 (0.0, 4.2)
**Self-Harm frequency**				
Every day	1 (3.8%)	0 (0.0%)	0 (0.0%)	1 (2.1%)
Twice a week	3 (11.5%)	2 (11.1%)	0 (0.0%)	5 (10.4%)
Once a week	4 (15.4%)	1 (5.6%)	1 (25.0%)	6 (12.5%)
Every two weeks	0 (0.0%)	4 (22.2%)	1 (25.0%)	5 (10.4%)
Once a month	3 (11.5%)	2 (11.1%)	0 (0.0%)	5 (10.4%)
3 monthly	3 (11.5%)	1 (5.6%)	1 (25.0%)	5 (10.4%)
Less often than three monthly	12 (46.2%)	7 (38.9%)	1 (25.0%)	20 (41.7%)
Missing	0 (0.0%)	1 (5.6%)	0 (0.0%)	1 (2.1%)
**Type of most recent self-harm**				
Ligature	2 (7.7%)	3 (16.7%)	0 (0.0%)	5 (10.4%)
Cutting	14 (53.8%)	11 (61.1%)	4 (100.0%)	29 (60.4%)
OD Medication/ Recreational drug overdose	6 (23.1%)	4 (22.2%)	0 (0.0%)	10 (20.8%)
Electrocution	1 (3.8%)	0 (0.0%)	0 (0.0%)	1 (2.1%)
Hunger strike	3 (11.5%)	0 (0.0%)	0 (0.0%)	3 (6.3%)
**How easy was it for you to get help?**				
Very Easy	6 (23.1%)	4 (22.2%)	0 (0.0%)	10 (20.8%)
Took some time	10 (38.5%)	6 (33.3%)	2 (50.0%)	18 (37.5%)
There was no help available	7 (26.9%)	4 (22.2%)	0 (0.0%)	11 (22.9%)
I didn't bother to ask	3 (11.5%)	3 (16.7%)	2 (50.0%)	8 (16.7%)
can’t remember	0 (0.0%)	1 (5.6%)	0 (0.0%)	1 (2.1%)

For peer review only - http://bmjopen.bmj.com/site/about/guidelines.xhtml

Appendix E: Table five coding variability across prison sites

Prison B	Prison A	Prison C	Prison D
**Prior to Nov 2013:** Not available<br>**Nov 2013 – Aug 2014:** coded as self-harm or concerns<br>**Aug 2014 – Oct 2015:** free text reasons<br>**Oct 2015 – present:**<br>1. Suicide attempt or statement of intent to take own life<br>2. Self-injury or statement to self-harm<br>3. Unusual behaviour/talk<br>4. Very low mood<br>5. Drug Alcohol Withdrawal<br>6. Other concerns<br>7. Self-harm warning received from court<br>*Not possible to distinguish acts of self-harm and suicide from intent, statements, or concerns.*	Recorded as free text.<br><br>*Not always possible to distinguish acts of self-harm and suicide from intent, statements, or concerns.*	Not available	**Prior to mid-2016:**<br>1. Suicide Attempt<br>2. Statement/thoughts of intent to kill self<br>3. Self-Harm<br>4. Statement of intent/thoughts to self-harm<br>5. Unusual Behaviour<br>6. Low mood<br>7. Problems related to Drug / Alcohol withdraw<br>8. External Concerns<br>9. Deportation<br>10. Bullying<br>11. Other<br>**Mid-2016 to present:**<br>1. Suicide attempt or Statement of intent to take own life<br>2. Self-Injury or Statement to Self-Harm<br>3. Unusual behaviour/Talk<br>4. Low Mood<br>5. Drug Alcohol Withdrawal<br>6. Other Concerns<br>*Method coding also provided and varies*
**Following coding:**<br>• 39% Related to SH or suicide<br>• 20% Other<br>• 41% Missing	**Following coding:**<br>• 36% due to SH incident or suicide attempt<br>• 39% related to SH or suicide<br>• 25% Other<br>• <1% Missing		**Following coding:**<br>• 39% due to a SH incident or suicide attempt<br>• 24% related to SH or suicide<br>• 33% Other<br>• 4% Missing

For peer review only

Appendix F: Figure 1 numbers of prisoners at unlock on last day of the month

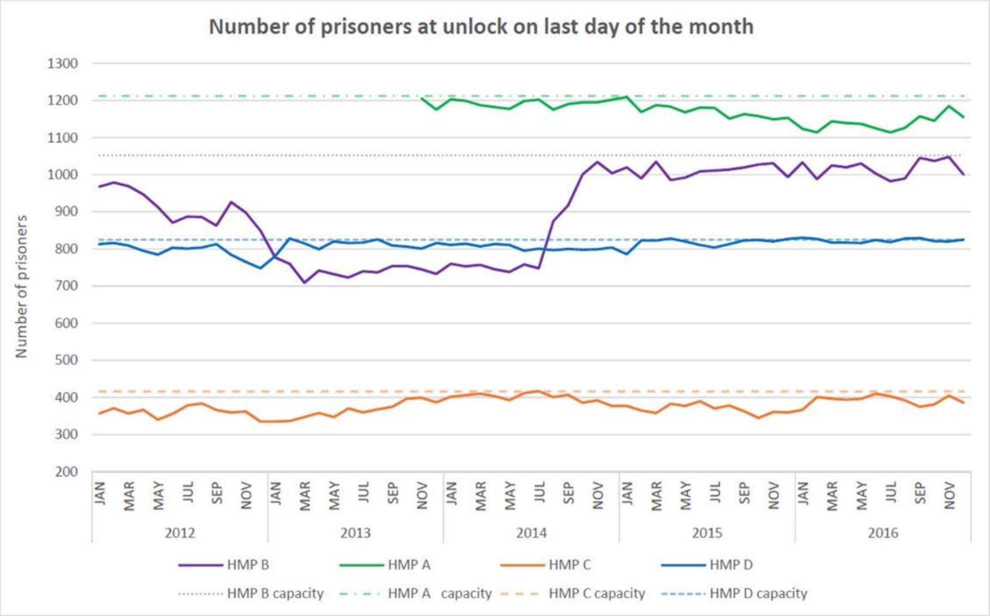

For peer review only - http://bmjopen.bmj.com/site/about/guidelines.xhtml

Appendix G: Table six standardised ACCT process costs

ACCT task per person	Initial ACCT opening and assignment of case manager by safer custody administration staff (minutes) £	Initial assessment by Case Manager (minutes) £	Case review attendance by two operational staff, one healthcare/other agency e.g., chaplaincy (minutes) £	Observation and case note entry into the ACCT documentation by Case manager (minutes) £	Post closure review (7 days after an ACCT has been shut). Interview between patient and Case Manager (minutes) £	Audit checks and data entry on the ACCT documentation once the ACCT shut by safer custody administrator (minutes) £
Time allocated	(30)	(30)	(60)	(5)	(30)	(30)
Standardised cost	4.60	6.50	39	1.05	6.50	4.60

For peer review only - http://bmjopen.bmj.com/site/about/guidelines.xhtml

Appendix H: Table seven training and implementation costs

Prison	Training Period	Number of staff trained	Number of training sessions	Cost for staff attending the training sessions (£)	Average cost per training sessions (£)]	Number of prisoners receiving the intervention	Overall intervention time (minutes)	Average intervention time spent per person (minutes)	Cost of intervention per head (£)	Cost of training and intervention delivery (£)	Overall cost per prisoner (£)]
HMP A	15.2.15-7.7.15	175	24	2625	172.87	26	1055	40.5	£35.17	6478	249.17
HMP B	15.2.15-7.7.15	175	24	2625	172.87	26	1055	40.5	£35.17	6478	249.17
HMP C	11.12.15-26.2.16	18	2	270	207.6	-	-	-	-	415.20	0
HMP D	23.6.16-9.8.16	8	2	120	132.6	4	90	22.5	£28.87	500.7	125.17

For peer review only - http://bmjopen.bmj.com/site/about/guidelines.xhtml

Appendix I: Figure 2 prisoner ACCTs, intervention and post assessment relative to baseline assessment

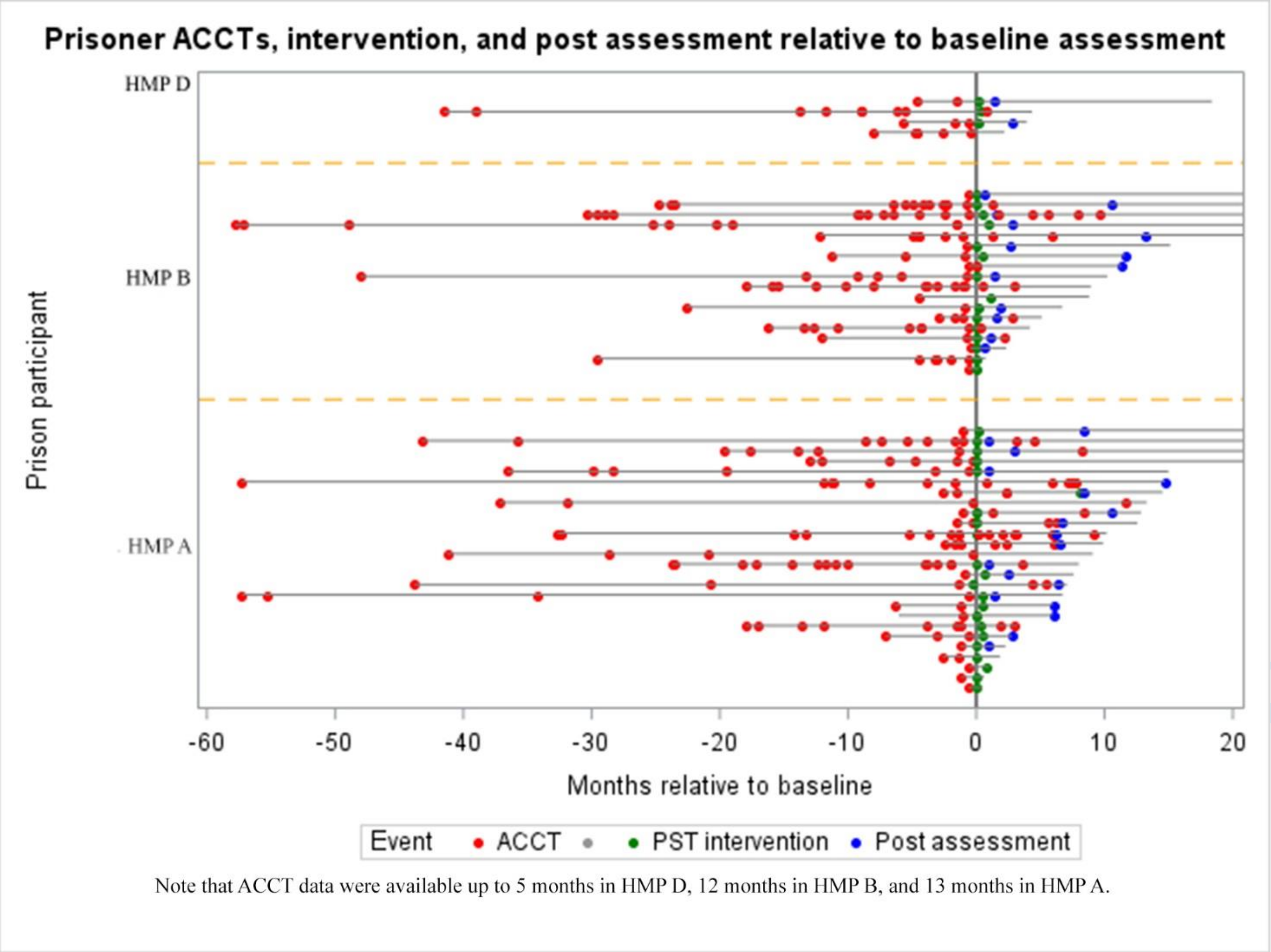

Appendix J: Figure 3 Phq-9 score at baseline and follow-up

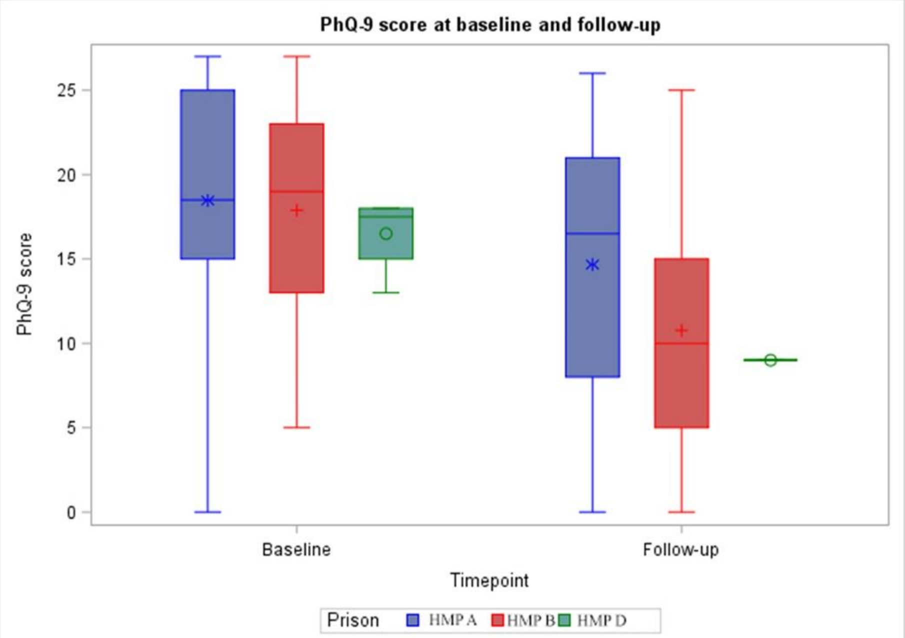

Note: follow-up took place a median of 2.8 months post-baseline, between 3 weeks and 15 months.

For peer review only - http://bmjopen.bmj.com/site/about/guidelines.xhtml
